# Identification and Characterization of Antiyeast Organic Acids Produced by *Lactiplantibacillus plantarum* 3121M0s Derived from Mongolian Traditional Fermented Milk, Airag

**DOI:** 10.3390/microorganisms13092017

**Published:** 2025-08-29

**Authors:** Md. Bakhtiar Lijon, Yuko Matsu-ura, Takumi Ukita, Kensuke Arakawa, Taku Miyamoto

**Affiliations:** 1 Graduate School of Environmental and Life Science, Okayama University, Okayama 7008530, Japan; 2 Faculty of Food Culture, Kurashiki Sakuyo University, Okayama 7100292, Japan; 3 Microbial Fermentation Research Center, Minori Co., Ltd., Okayama 7011221, Japan; 4 Functional Food Creation Research Institute Co., Ltd., Okayama 7161241, Japan

**Keywords:** lactic acid bacteria, yeast, organic acid, acetic acid, lactic acid, 3-phenyllactic acid, *Lactiplantibacillus plantarum*, fermented food, food spoilage, biopreservation

## Abstract

Lactic acid bacteria are beneficial for food biopreservation by inhibiting not only bacteria but also fungi. However, reports on the control of fungi, especially yeasts, by lactic acid bacteria are limited. In this study, strain 3121M0s derived from Mongolian traditional fermented milk, airag, was selected with relatively high antiyeast activity among 236 strains, and identified as *Lactiplantibacillus plantarum*. The activity was exhibited under acidic conditions and remained stable after heating. It was also highly resistant to catalase and proteases, indicating that the primary antiyeast substances of 3121M0s were neither H_2_O_2_ nor peptides. Then, organic acids (lactic acid, acetic acid, 4-hydroxyphenyllactic acid, 4-hydroxybenzoic acid, and 3-phenyllactic acid) were detected and quantified in the ethyl acetate extract of the 3121M0s culture supernatant. Among them, only acetic acid showed antiyeast activity on its own, and the activity was enhanced by lactic acid or 3-phenyllactic acid. Compared to the type strain of *L. plantarum*, the production of lactic acid from 3121M0s was almost equal, but acetic acid and 3-phenyllactic acid were about 1.5 times higher. These results suggest that strain 3121M0s would be useful as a biopreservative starter for fermented foods susceptible to yeast contamination due to being produced in open environments without final sterilization.

## 1. Introduction

Microbial contamination of food poses health risks as well as leads to economic losses and social issues such as food waste. Biological food contaminants are primarily bacteria, but fungi should also be given careful attention because they also cause food poisoning and spoilage [1,2]. From the perspective of preventing food poisoning, it is necessary to control fungi, particularly molds such as some *Aspergillus* spp. and *Fusobacterium* spp., which have the potential to produce mycotoxins such as aflatoxins and fumonisins [3,4,5].

Another major fungus, yeast, has long been used in the production of various fermented foods since ancient times. The most common species is *Saccharomyces cerevisiae*, and other known species include *Debaryomyces hansenii*, *Kluyveromyces marxianus*, *Pichia kudriavzevii*, *Wickerhamomyces anomalus*, and *Zygosaccharomyces rouxii*; most of which are considered safe [6,7,8]. However, these yeasts are often reported to be involved in food spoilage, and therefore should be controlled when undesirable [6,8,9,10,11,12,13,14,15,16,17,18,19]. In addition, although there are very few cases of food poisoning caused by yeasts, they can cause foodborne illness such as candidiasis by *Candida albicans* and its relatives [8,20]. Other species, including *Rhodotorula mucilaginosa* and even *S. cerevisiae,* have also been reported to rarely cause infection through food, particularly in immunocompromised individuals [21,22].

Yeasts associated with food are generally sensitive to heat, but relatively resistant to moderately low pH, low water activity, and osmotic stress such as high salt and sugar concentrations [15,16,23]. Therefore, yeasts are prone to deteriorating foods, even if they have been fermented, concentrated, and salted. This is particularly noticeable in foods produced under open environments without final heat treatment, because undesirable yeasts can easily contaminate and survive in such products. Dairy products such as cheese and traditional fermented milk are prime examples [6,9,10,12,13,14,17,18,19,24,25,26,27].

To effectively inhibit yeasts, chemical food additives such as calcium sorbate, sodium benzoate, calcium propionate, and sulfur dioxide are commonly used [28,29]. However, consumers have increasingly avoided synthetic preservatives, and a food preservation method using naturally derived preservatives with a long history of safe consumption, known as biopreservation and biopreservatives, has been gaining attention [29,30,31,32]. In particular, the use of lactic acid bacteria (LAB), which are safe, familiar to humans, and possess high antimicrobial effects, is anticipated for food biopreservation. LAB biopreservatives have been studied for their effectiveness mainly against bacteria, and there are also various reports against molds in dairy products, bread, grains, fruits, and vegetables. However, reports on yeasts are comparatively limited. Furthermore, most of these reports focus on LAB screening and food application tests, and a few have examined the details of antiyeast substances. To date, acetic acid (AA), 3-phenyllactic acid (PLA), hydroxy fatty acids, 3,5-dicaffeoylquinic acid, reuterin, cyclic dipeptides, bacteriocin, and proteinaceous compounds, including bacteriocin-like inhibitory substances, have been reported as reliable antiyeast substances [33,34,35,36,37,38,39,40,41,42].

By the way, LAB inhabit various environments, and traditional fermented dairy products are one of the most representative sources for isolating useful LAB strains. Mongolian traditional fermented milk, airag, which is produced by natural fermentation or by backslopping from previous batches without adding any commercial starter LAB strains, has been found to have health benefits. In recent years, its fermentation microbiota has been increasingly characterized using culture-dependent [43,44,45,46,47] and culture-independent methods [48,49]. In the references, it is shown that the main fermentation microorganisms in airag were LAB, and the most abundant species was consistently *Lactobacillus helveticus*. Other LAB species, such as *Lentilactobacillus kefiranofaciens*, *Lentilactobacillus parakefiri, Lactococcus lactis*, *Lacticaseibacillus casei*, *Lactiplantibacillus plantarum*, *Leuconostoc mesenteroides*, and *Streptococcus thermophilus*, and yeast species such as *K. marxianus*, *Kazachstania unispora*, *S. cerevisiae*, *Issatchenkia orientalis*, and *Pichia manshurica,* were frequently detected as well. In addition, some LAB strains isolated from airag have been characterized as potential contributors to its beneficial effects in fermentation and health promotion [50,51,52,53]. Furthermore, LAB strains producing bacteriocins have also been isolated and characterized [39,54,55,56,57], and their potential applications extend beyond fermentation starters and probiotics to biopreservation in food.

This study aimed to select a strain with relatively high antiyeast activity from our LAB library containing strains derived from airag, and to identify and characterize antiyeast substances produced by the strain. LAB with antiyeast activity are expected to be used effectively as starter cultures for fermented foods, especially produced under open environments without final heat treatment, such as cheese and traditional fermented milk [17]. Stated differently, antiyeast LAB strains could serve as biopreservative starters.

## 2. Materials and Methods

### 2.1. Microbial Strains and Culture Conditions

A total of 236 LAB strains, which were formerly isolated from various animals, plants, and traditional fermented foods and stored in our laboratory, were used for screening based on their antiyeast activity. The LAB strains were cultivated in de Man, Rogosa, and Sharp (MRS) broth (Oxoid; Thermo Fisher Scientific Inc.; Waltham, MA, USA) at their respective optimal temperatures (30 or 37 °C) for 24–48 h before use. Eleven strains of food spoilage yeasts (Table 1 and Table 2) used as indicators in the antiyeast activity assay were cultivated in Yeast and Mold (YM) broth (Difco; Becton, Dickinson and Company; Franklin Lakes, NJ, USA) at 25 °C for 48 h. All culture media except reconstituted skim milk (RSM) were sterilized at 121 °C for 15 min. RSM was prepared using 10% (*w*/*v*) skim milk powder (Megmilk Snow Brand Co., Ltd.; Sapporo, Hokkaido, Japan) and autoclaved at 110 °C for 20 min.

### 2.2. Antiyeast Activity Assay

The overlay method described by Magnusson and Schnürer (2001) [42], with a few modifications, was used primarily to screen for the 236 LAB strains with their antiyeast activity. Each LAB strain culture was centrifuged at 1600× *g* for 20 min to collect the cell suspension by removing the supernatant, and then 5 µL of the suspension was inoculated onto a plate of MRS agar (Thermo Fisher Scientific Inc.), followed by incubation at 30 °C for 48 h under anaerobic conditions using AnaeroPack (Sugiyama-Gen; Tokyo, Japan). The MRS plate was then overlaid with 10 mL of YM agar (Becton, Dickinson and Company) containing 1.0 × 10^4^ CFU/mL of an indicator yeast strain. After solidification of the agar, the plate was incubated at 25 °C for 48 h. The antiyeast activity was evaluated by the size of the inhibitory zone formed around the inoculated LAB colony.

The strains selected by the overlay assay were further screened for their antiyeast activity using the agar–well diffusion method [42]. Before the assay, samples were prepared as follows. Each LAB strain culture was centrifuged (1600× *g*, 20 min), and filtrated with a 0.45 µm filter (Membrane Solutions, LLC; Auburn, WA, USA) to make cell-free culture supernatant (CFS). Next, CFS was lyophilized and then dissolved in citrate-phosphate buffer (pH 4.0) to make 10× tenfold concentrated CFS (10× CFS). In the assay, an indicator strain culture was inoculated into 20 mL of YM agar at a density of 1.0 × 10^4^ CFU/mL, and then the agar was poured into a plate. After solidification, 6 mm diameter wells were made in the agar plate, and then 50 µL of 10× CFS was added into each well. After that, the plate was incubated at 25 °C for 48 h. The antiyeast activity was evaluated by the size of the inhibitory zone formed around the well.

Following the same procedure as the agar–well diffusion method described above, the antiyeast activity of 10× CFS (pH 4.0) of a control strain and organic acids (OAs) contained in 10× CFS was also assayed against *R. mucilaginosa* JCM 8115^T^. The OA solution was adjusted to pH 4.0 using 1–6 M NaOH and HCl before the assay.

### 2.3. Cocultivation in Skim Milk

The antiyeast effect of the selected LAB strain, 3121M0s, was verified by cocultivation with a food spoilage yeast strain, *R. mucilaginosa* JCM 8115^T^, in RSM. The LAB and yeast strains were inoculated into RSM together at 5–6 × 10^7^ and 1 × 10^2^ CFU/mL, respectively. As a control, RSM inoculated with either 3121M0s or JCM 8115^T^ was used. After inoculation, RSM was incubated at 25 °C for 72 h, and culture pH and viable cell counts were measured at 0, 12, 24, 48, and 72 h incubation. The viable cell counts of the LAB and yeast strains were measured using cultivation in MRS agar supplemented with 10 ppm of cycloheximide and YM agar with 100 ppm of chloramphenicol at 30 and 25 °C for 48 h, respectively.

### 2.4. Species Identification

To identify the species of the selected strain, 3121M0s, its 16S rRNA gene was amplified using PCR with 27F (5′-AGAGTTTGATCMTGGCTCAG-3′) and 1525R (5′-AAGGAGGTGATCCAGCC-3′) primers [58], and sequenced. Total DNA of the strain was extracted and purified as described by Klaenhammer (1993) [59]. For the PCR, KAPA Taq EXtra HotStart ReadyMix (Kapa Biosystems, Inc.; Wilmington, MA, USA) and SimpliAmp Thermal Cycler (Applied Biosystems; Waltham, MA, USA) were used. The PCR conditions were followed according to the reference. The resulting PCR amplicon was extracted using MagExtractor DNA Fragment Purification Kit (Toyobo Co., Ltd.; Osaka, Japan) after agarose gel electrophoresis, and then submitted to the DNA sequencing service at Eurofins Genomics K.K. (Tokyo, Japan). The obtained 16S rRNA gene sequence was analyzed using the Basic Local Alignment Search Tool (BLAST; Ver. 2.16.0) of the National Center for Biotechnology Information (NCBI). Then, it was used to construct a phylogenetic tree with 16S rRNA gene sequences of the type strains of all species of the genus *Lactiplantibacillus* and the outgroup *Lactobacillus delbrueckii* subsp. *bulgaricus* and *Escherichia coli*, after trimming to align the lengths using the Gblocks program (Ver. 0.91b) [60]. The tree was constructed by the maximum-likelihood method with the Jukes–Cantor model using the CLC Main Workbench software (Ver. 6.9.2; Qiagen N.V.; Venlo, The Netherlands).

Since it is impossible to identify the species of *L. plantarum*-group LAB based on only 16S rRNA gene sequences, an additional multiplex PCR [61] concerning the species-specific *recA* gene was performed. Four reference strains, *L. plantarum* JCM 1149^T^, *Lactiplantibacillus argentoratensis* JCM 16169^T^, *Lactiplantibacillus paraplantarum* JCM 12533^T^, and *Lactiplantibacillus pentosus* JCM 1558^T^, and four PCR primers, paraF (5′-GTCACAGGCATTACGAAAAC-3′), pentF (5′-CAGTGGCGCGGTTGATATC-3′), planF (5′-CCGTTTATGCGGAACACCTA-3′), and pREV (5′-TCGGGATTACCAAACATCAC-3′), were used. The PCR conditions were followed according to the reference, and the resulting amplicons were profiled by electrophoresis to identify the species.

After the species identification, the obtained 16S rRNA sequence was submitted to the DNA Data Bank Japan (DDBJ) to obtain an accession number (LC884927).

### 2.5. Determination of Optimal Culture Conditions

To determine the optimal culture conditions of the selected LAB strain, 3121M0s, the bacterial growth was evaluated by sequentially measuring the culture pH at 0, 4, 8, 12, 16, 18, 24, 36, 48, and 72 h of incubation in MRS broth at 25, 30, and 37 °C. The culture turbidity (at OD 620 nm) and antiyeast activity were also measured at 0, 4, 8, 12, 18, 24, 36, 48, and 72 h of incubation at 30 °C. The antiyeast activity was assayed using the agar–well diffusion method with 10× CFS samples against *R. mucilaginosa* JCM 8115^T^.

### 2.6. Evaluation of Effects of pH, Heating, and Enzymes on Antiyeast Activity

To evaluate the effect of pH on the antiyeast activity of the selected strain, 3121M0s, the lyophilized CFS was dissolved in hydrochloric acid-potassium chloride buffer (pH 2.0), in citrate-phosphate buffer (pH 3.0, 4.0, 5.0, 6.0, 7.0, and 8.0), and carbonate-bicarbonate buffer (pH 9.0 and 10.0) to prepare each 10× CFS, followed by filtration through a 0.45 µm filter. The 10× CFS (pH 2.0–10.0) were used for the agar–well diffusion antiyeast activity assay against *R. mucilaginosa* JCM 8115^T^.

Next, to evaluate the effect of heating, the 10× CFS (pH 4.0) was treated at 95 °C for 10 min and 121 °C for 15 min. Then, it was used for the activity assay in the same manner as above.

Moreover, to evaluate the effects of enzymatic treatments, 1 mL of CFS of the selected strain, 3121M0s, was reacted with 5 mg of 47,031 U/mg catalase (pH 7.0, 25 °C; Sigma Aldrich; St. Louis, MO, USA), 1650 U/mg pepsin (pH 2.0, 37 °C; Sigma-Aldrich), 1300 U/mg trypsin (pH 8.0, 37 °C; Fujifilm Wako Pure Chemical Corporation; Osaka, Japan), 66 U/mg α-chymotrypsin (pH 7.8, 25 °C; Nacalai Tesque, Kyoto, Japan) and 38.6 U/mg proteinase K (pH 7.5, 37 °C; Nacalai Tesque) for 1 h. After the reaction, the CFS was inactivated by heating at 95 °C for 10 min. Then, each 10× CFS (pH 4.0) was prepared and assayed for the antiyeast activity in the same manner as above.

### 2.7. Ethyl Acetate Extraction

CFS of the selected strain, 3121M0, and the control strain, JCM 1149^T^, were adjusted to pH 2.0 using 1–6 M HCl, and then mixed well with ethyl acetate (Fujifilm Wako Pure Chemical) at a ratio of 1:2 in a separating funnel. After leaving them for 15 min, the upper layer (organic fraction) was collected and further mixed well with ethyl acetate again. This step was repeated three times. After that, ethyl acetate solvent was removed using a rotary evaporator (Tokyo Rikakikai Co., Ltd.; Tokyo, Japan), and the concentrated extracts were diluted to one-tenth the volume of the original CFS using citrate-phosphate buffer (pH 4.0). The extracts obtained here were also assayed using the agar–well diffusion method against *R. mucilaginosa* JCM 8115^T^.

### 2.8. HPLC and MS

To separate OA, the ethyl acetate extracts obtained above were subjected to reverse-phase HPLC (Shimadzu Corporation, Kyoto, Japan) with a YMC Pack ODS-AM column (4.6 mm × 250 mm; YMC Co., Ltd.; Kyoto, Japan) at 30 °C. Elution was performed with aqueous acetonitrile containing 0.05% (*v*/*v*) trifluoroacetic acid at a flow rate of 1 mL/min. The gradient program for the elution was as follows: 0–5 min, 5% (*v*/*v*) acetonitrile; 5–40 min, 5 to 90% (*v*/*v*) acetonitrile; and 40–45 min, 90% (*v*/*v*) acetonitrile. The eluate was monitored at a wavelength of 220 nm. As analytical standards for qualification and quantification, special grade reagents of AA (Nacalai Tesque), benzoic acid (Fujifilm Wako Pure Chemical), caffeic acid (Tokyo Chemical Industry Co., Ltd.; Tokyo, Japan), 4-hydroxybenzoic acid (OHBA; Tokyo Chemical Industry), 4-hydroxyphenyllactic acid (OHPLA; Tokyo Chemical Industry), 3-(4-hydroxyphenyl)propionic acid (Tokyo Chemical Industry), DL-lactic acid (LA; Nacalai Tesque), L-malic acid (Nacalai Tesque), PLA (Tokyo Chemical Industry), and succinic acid (Nacalai Tesque) were used.

LA was further distinguished between D- and L-forms using chiral reverse-phase HPLC with a Sumichiral OA-5000 column (4.6 mm × 150 mm; Sumika Chemical Analysis Service, Ltd.; Osaka, Japan) at 25 °C. Elution was isocatically performed with 5% (*v*/*v*) 2-propanol containing 2 mM copper sulfate at a flow rate of 1 mL/min. The eluate was monitored at a wavelength of 254 nm. As analytical standards for qualification and quantification, special grade reagents of D- and L-LA (Tokyo Chemical Industry) were used.

The OAs separated by HPLC and tentatively identified based on their retention times were further reliably identified based on their molecular weight information obtained using a mass spectrometer, JMS-700N (JEOL Ltd.; Tokyo, Japan), in the Common Facilities Division of the Office for Research Initiative and Development at Nagasaki University, Japan. Among the OA, only LA was analyzed by the positive-ion mode of fast atom bombardment mass spectrometry (FAB-MS) with glycerol as a matrix. The other OAs were analyzed by electron ionization mass spectrometry (EI-MS).

### 2.9. Evaluation of Effects of Adding Tyrosine and Phenylalanine into Culture Medium

It is known that the addition of L-tyrosine (Tyr) and L-phenylalanine (Phe) to the medium increases the production of OHPLA and PLA by LAB, respectively [62]. To evaluate the effects of the two amino acids on OHPLA and PLA production and the antiyeast activity of the 10× CFS, modified MRS broth supplemented with 0.4% Tyr and/or Phe was used to cultivate the selected strain, 3121M0s. After cultivation at 30 °C for 48 h, the CFS and 10× CFS (pH 4.0) were prepared. Then, the CFS was analyzed to quantify the content of OHPLA and PLA, and the 10× CFS was used for the antiyeast activity assay against *R. mucilaginosa* JCM 8115^T^ in the same manner as above.

### 2.10. Statistical Analysis

Data are presented as the mean ± standard deviation. The data were statistically evaluated using a two-way analysis of variance (ANOVA) with Tukey’s test in IBM SPSS Statistics 20.0. Differences were considered significant at *p* < 0.05.

## 3. Results

### 3.1. Selection of Strain 3121M0s with High Antiyeast Activity

As the first screening, 236 LAB strains were assayed for their antiyeast activity using the overlay method against the three indicator yeast strains. Among them, four strains (3121M0s, 3272B0, 3273B0, and 3461M0), which were formerly isolated from Mongolian traditional fermented milk, airag [56], showed relatively high activity against the two food spoilage yeast strains, *Rhodotorula mucilaginosa* JCM 8115^T^ and *Candida parapsilosis* JCM 1612^T^ (Table 1). The four strains were next subjected to the antiyeast activity assay using the agar well diffusion method. As a result, only strain 3121M0s showed antiyeast activity against *R. mucilaginosa* JCM 8115^T^ (Table 1), and, therefore, this strain was selected for further experiments.

The selected strain, 3121M0s, was assayed for the antiyeast activity against the other eight strains of food spoilage yeasts. The strain exhibited activity against the five and four yeast strains on the overlay and agar–well diffusion methods, respectively (Table 2). The activity was particularly high against the type strain of *Debaryomyces hansenii,* generally with high tolerance to osmotic stress, similar to that against *R. mucilaginosa*.

### 3.2. Evaluation of Antiyeast Effect of Strain 3121M0s in Skim Milk

**Figure 1:**
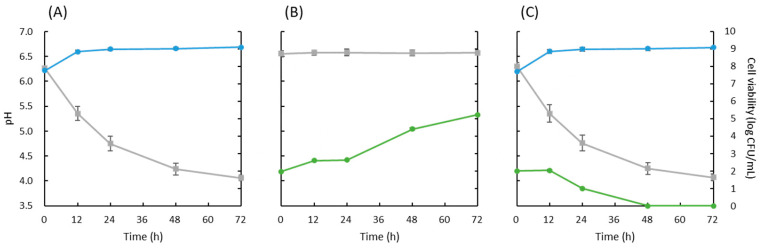
Changes in culture pH (gray), and cell viability of strain 3121M0s (light blue) and *Rhodotorula mucilaginosa* JCM 8115^T^ (yellow green) during mono- (**A**,**B**) or co-cultivation (**C**) in reconstituted skim milk at 25 °C for 72 h.

To verify the antiyeast effect of strain 3121M0s in actual food, cocultivation with *R. mucilaginosa* JCM 8115^T^ was performed in RSM. As a result, strain 3121M0s inhibited the growth of *R. mucilaginosa* while maintaining its own acid production or growth (Figure 1).

### 3.3. Species Identification of Strain 3121M0s

To identify the species of strain 3121M0s, its 16S rRNA gene was sequenced. The resulting sequence (LC884927) had almost 100% identity to strains of the *L. plantarum*-group LAB. A similar result was also observed in a phylogenetic tree constructed based on the 16S rRNA gene sequences of strain 3121M0s and the type strains of all species of the genus *Lactiplantibacillus* (Figure S1). Next, for further identification, the species-specific multiplex PCR was performed. The resulting electrophoretic profile shown in Figure S2 concluded that strain 3121M0s was identified as *L. plantarum*.

### 3.4. Determination of Optimal Culture Conditions of Strain 3121M0s

To determine the optimal incubation temperature for strain 3121M0s, the culture turbidity was measured sequentially during cultivation at 25, 30, and 37 °C for 72 h. The growth at 30 °C was faster and higher than at 25 and 37 °C, respectively (Figure S3). From the result, 30 °C was selected as the incubation temperature for subsequent experiments.

**Figure 2:**
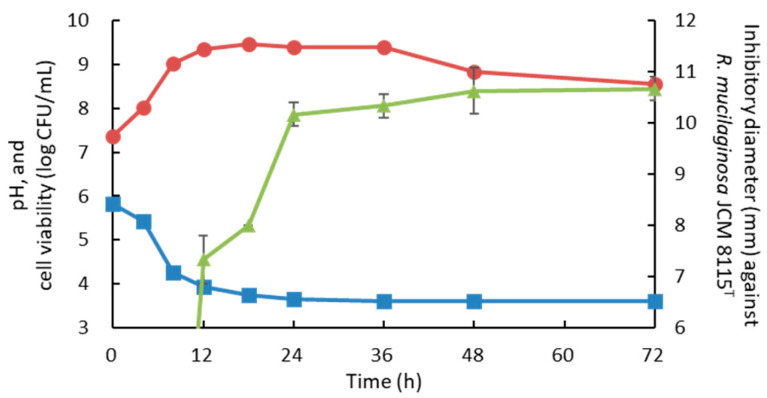
Changes in culture pH (light blue), cell viability (red), and antiyeast activity against *Rhodotorula mucilaginosa* JCM 8115^T^ (yellow green) of strain 3121M0s cultivated in MRS broth at 30 °C for 72 h.

Culture pH and cell viability at 30 °C were also measured with the antiyeast activity of 10× CFS of strain 3121M0s against *R. mucilaginosa* JCM 8115^T^ to determine the optimal incubation time. The growth peaked at 12–36 h, but the activity was relatively higher at 24–72 h (Figure 2). Therefore, the subsequent incubation time was set to 48 h.

### 3.5. Evaluation of pH, Heating, and Enzymes on Antiyeast Activity of Strain 3121M0s

**Figure 3:**
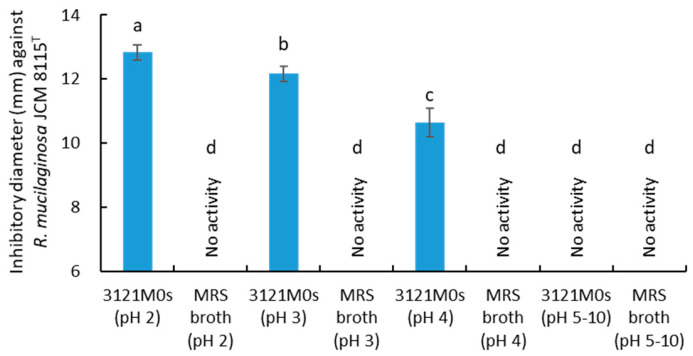
Effect of pH (2–10) on antiyeast activity against *Rhodotorula mucilaginosa* JCM 8115^T^ of strain 3121M0s. Different lowercase letters denote a significant difference (*p* < 0.05).

To evaluate the effect of pH on the antiyeast activity of strain 3121M0s, the activity assay of the 10× CFS was performed against *R. mucilaginosa* JCM 8115^T^ under pH 2.0–10.0. The activity was observed at pH 2.0–4.0, not pH 5.0–10.0 (Figure 3). No activity was also observed in 10× MRS broth at pH 2.0–10.0 used as a control.

**Figure 4:**
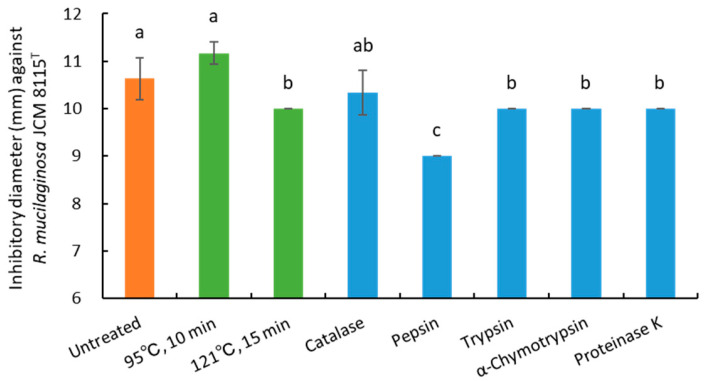
Effects of heating (121 °C, 15 min) and enzymatic treatment (catalase and four proteases) on antiyeast activity against *Rhodotorula mucilaginosa* JCM 8115^T^ of strain 3121M0s. Different lowercase letters denote a significant difference (*p* < 0.05).

Next, the effect of heating (95 °C for 10 min and 121 °C for 15 min) was evaluated. As a result, 121 °C for 15 min slightly reduced the activity, but 95 °C for 10 min was unaffected (Figure 4).

Then, the effects of catalase and proteases were also evaluated. Catalase had no observable effect, while trypsin, α-chymotrypsin, and proteinase K, among the proteases, slightly reduced the activity (Figure 4). In addition, pepsin markedly reduced the activity, but sufficient intensity remained after the reaction. These results meant that the primary antiyeast substances of strain 3121M0s were neither H_2_O_2_ nor peptides.

### 3.6. Identification and Quantification of Organic Acids Produced by Strain 3121M0s

Antiyeast substances produced by strain 3121M0s were separated using ethyl acetate extraction. The antiyeast activity was strongly detected in the organic fraction (upper layer), while weakly detected in the aqueous fraction (lower layer). The organic fraction was subjected to HPLC after evaporation, followed by redissolving for further analysis.

**Figure 5:**
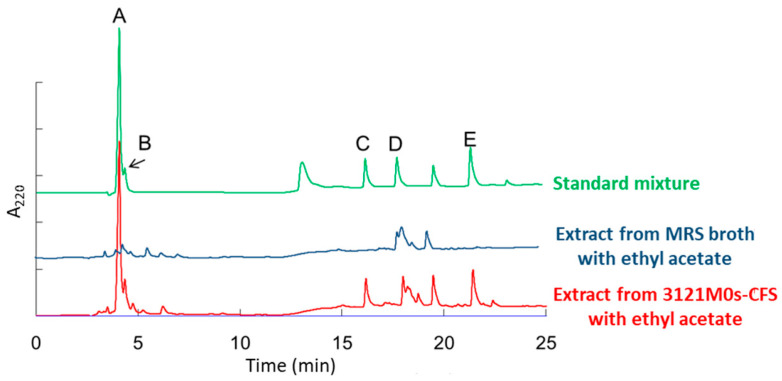
HPLC chromatogram of extract from the cell-free culture supernatant of strain 3121M0s with ethyl acetate. MRS broth extract was used as a control. Standards: (A) DL-lactic acid; (B) acetic acid; (C) 4-hydroxyphenyllactic acid; (D) 4-hydroxybenzoic acid; and (E) 3-phenyllactic acid.

**Figure 6:**
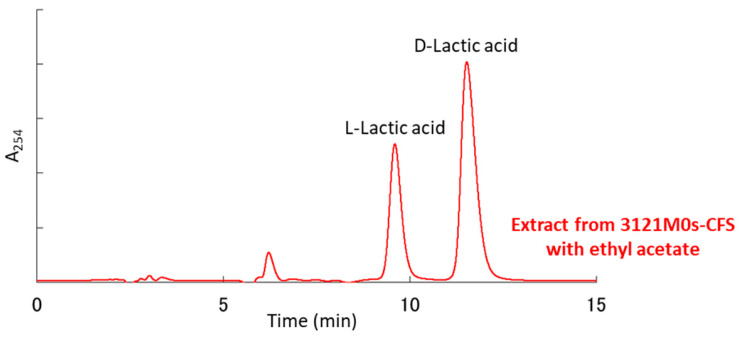
Chiral HPLC chromatogram of extract from the cell-free culture supernatant of strain 3121M0s with ethyl acetate. Reagents D-and L-lactic acids were used as standards.

In the HPLC analysis, several peaks were detected, and five OAs (LA, AA, OHPLA, OHBA, and PLA) were roughly identified based on their retention times compared to those of the standard chemicals (Figure 5). More reliable identification was achieved using EI- and FAB-MS (Figure S4). In addition, the chirality (D- and L-forms) of LA was identified using another HPLC (Figure 6). Then, the concentrations of the five OAs and the two isomers were calculated from the HPLC peak areas obtained. In the CFS of strain 3121M0s, LA (16,053.0 mg/L = 178 mM) was the most abundant OA, and the ratio of the D- and L-forms was 2:1 (Table 3). The second most abundant OA was AA (1437.9 mg/L = 23.9 mM), followed by PLA (99.1 mg/L = 0.60 mM), OHBA (26.9 mg/L = 0.195 mM), and OHPLA (28.7 mg/L = 0.158 mM).

### 3.7. Antiyeast Activity of Organic Acids Produced by Strain 3121M0s

Based on the results in Section 3.6 and Table 3, the concentrations of each OA in the 10× CFS were calculated, and the antiyeast activity of the individual and mixed OA solutions was assayed against *R. mucilaginosa* JCM 8115^T^. The total OA mixture in the 10× CFS exhibited slightly lower activity than that of the whole 10× CFS, but the difference was not so large (Table 4).

**Figure 7:**
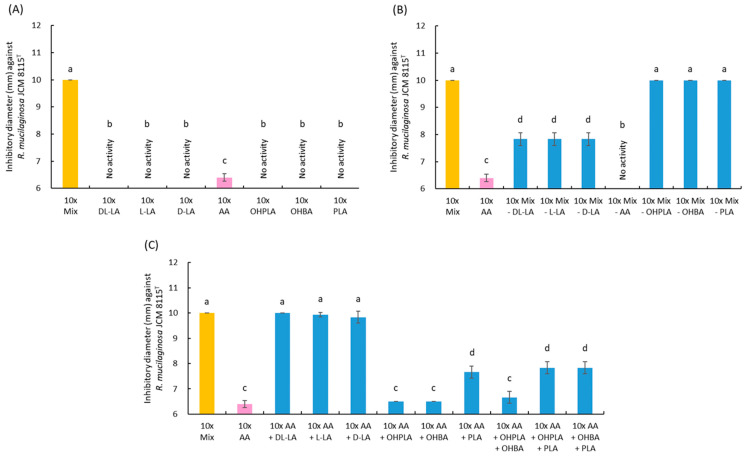
Antiyeast activity of the tenfold concentrated solutions (pH 4.0) of single (**A**) and mixed (**B**,**C**) organic acids contained in the cell-free culture supernatant of strain 3121M0s. (**B**) The activity of tenfold mixtures of all organic acids except one. (**C**) The activity of tenfold mixtures containing acetic acid and another organic acid. The antiyeast activity was measured against *Rhodotorula mucilaginosa* JCM 8115^T^. Used organic acids: LA, lactic acid; AA, acetic acid; OHPLA, 4-hydroxyphenyllactic acid; OHBA, 4-hydroxyphenylbenzoic acid; PLA, 3-phenyllactic acid; and Mix, mixture of these organic acids. Different lowercase letters denote a significant difference (*p* < 0.05).

In the assay with the individual OA, only AA showed low activity, while no activity was observed with the other OA (Figure 7A). Next, the antiyeast activity of the mixtures excluding one OA was assayed. The mixtures, excluding AA or LA, completely or partially lost the activity, respectively (Figure 7B). Exclusion of OHPLA, OHBA, or PLA had no observable effect on the activity. Moreover, a combination of AA and another OA was tested. The combination of AA and LA exhibited complete activity equal to the total OA mixture (Figure 7C). The combination of AA and PLA showed moderate activity, and OHPLA and PHBA had no observable effect on the activity of AA. These results indicated that the main antiyeast substance produced by 3121M0s was AA, and that LA and PLA enhanced the activity of AA. In addition, there was no difference in effect between D- and L-forms of LA (Figure 7B,C).

### 3.8. Comparison of Organic Acid Productivity and Antiyeast Activity Between Strains 3121M0s and JCM 1149^T^

To verify the superiority of strain 3121M0s in terms of OA productivity and antiyeast activity, it was compared with the type strain (JCM 1149^T^) of *L. plantarum*. Strain JCM 1149^T^ showed nearly identical levels (16,244.4 mg/L = 180 mM) of LA production to 3121M0s, and their D/L-form ratio was also similar, at approximately 2/1 (Table 3). However, the production levels of AA (902.2 mg/L = 15.0 mM), OHPLA (3.0 mg/L = 0.016 mM), and PLA (69.2 mg/L = 0.42 mM) in JCM 1149^T^ were clearly lower than those in 3121M0s. Conversely, the production of OHBA was higher (34.4 mg/L = 0.249 mM) in JCM 1149^T^. Regarding antiyeast activity, the 10× CFS and the total OA mixture of JCM 1149^T^ were significantly inferior to those of 3121M0s (Table 4). Thus, strain 3121M0s had superior antiyeast activity, as well as AA, OHPLA, and PLA productivity, compared to strain JCM 1149^T^.

### 3.9. Effects of L-Tyrosine and L-Phenylalanine on Organic Acid Productivity and Antiyeast Activity of Strain 3121M0s

The effects of adding Tyr and Phe into the culture broth on the OHPLA and PLA production from strain 3121M0s were evaluated. The addition of Tyr increased the OHPLA production (28.7 to 125.4 mg/L = 0.688 mM) but significantly decreased the PLA production (99.1 mg/L to trace) (Table 5). Conversely, the addition of Phe increased the PLA production (99.1 to 126.5 mg/L = 0.76 mM), but inhibited the OHPLA production (28.7 mg/L to trace). Furthermore, the co-addition of both amino acids suppressed the increased OHPLA production with Tyr alone (125.4 to 84.9 mg/L), and reduced the PLA production even compared to the control without adding them (99.1 to 88.9 mg/L). Although such changes in the OHPLA and PLA production were observed with the addition of Tyr and Phe, no significant changes were observed in the antiyeast activity against *R. mucilaginosa* JCM 8115^T^ of their 10× CFS.

## 4. Discussion

With the aim of developing a useful antiyeast biopreservative starter for fermented foods, strain 3121M0s with comparatively high activity was selected from 236 strains in our LAB library (Table 1). Although the strain exhibited no inhibitory effects against *I. orientalis*, *P. kudriavzevii*, and *W. anomalus*, it showed low antiyeast activity against each type strain of *C. parapsilosis* and *S. cerevisiae*, and pronounced activity against *R. mucilaginosa*, *D. hansenii*, *Hanseniaspora uvarum*, *K. marxianus*, and *Z. rouxii* (Table 1 and Table 2). The relatively substantial inhibition observed particularly against *R. mucilaginosa* and *D. hansenii* aligns with previous reports highlighting their high sensitivity to antiyeast LAB [63,64,65,66,67]. Many studies have consistently reported that the main yeast contaminants in raw milk and non-fermented dairy products, such as cream and butter, were overwhelmingly *Candida* spp. [9,12,13,14]. However, in traditional fermented milk and cheese, *Candida* spp. were not the only prevalent yeasts; rather, *D. hansenii*, *K. marxianus,* and *Kluyveromyces lactis* were commonly detected at high abundance and considered to be the primary spoilage yeasts [6,9,10,14,17,18,19,24,25]. Also, in airag, *K. marxianus* was reported to be the most dominant yeast [45]. Although *Rhodotorula* spp. are generally non-dominant yeasts, they have been widely detected in raw milk and non-fermented and fermented dairy products [6,9,10,12,13,14,17,18,19,24,25]. In addition, *Rhodotorula* spp. are known to deteriorate the appearance and flavor of products due to their reddish-pink color and high lipolytic activity [9,13]. Figure 1 shows that strain 3121M0s completely inhibited 10^2^ CFU/mL of *R. mucilaginosa* cocultivated in RSM. The effect was notably stronger than previously reported results of the other antiyeast LAB [63,66,67]. It is known that the number of contaminating yeast in traditional fermented milk and cheese can often reach 10^4^ CFU/mL or more, but the yeast counts in raw milk and dairy products in the early fermentation stages are less than 10^2–3^ CFU/mL [6,9,18,19,24,25,66,67]. Therefore, the significant inhibitory effects not only on *R. mucilaginosa* but also on *D. hansenii* and *K. marxianus* in the agar–well diffusion assay (Table 1 and Table 2), and the inhibition on *R. mucilaginosa* in the cocultivation test (Figure 1) demonstrate a high practical potential of strain 3121M0 for biopreservation in fermented dairy products.

The species of strain 3121M0s was identified as *L. plantarum* (Supplemental Figures S1 and S2). *L. plantarum* is one of the most versatile LAB species with ecological and metabolic adaptability based on its larger genome and higher genetic diversity compared to other LAB species, and inhabits a wide range of ecological niches, including plants, meats, the mammalian gastrointestinal tract, and fermented foods, including traditional products such as airag [68,69,70,71]. Due to such versatility, *L. plantarum* strains are used as safe and highly functional starter and probiotic cultures in dairy, meat, and many plant-based fermentations such as Feta cheese, dry sausage, sauerkraut, pickles, sourdough, and kimchi. In fact, various fermentation starter cultures, including *L. plantarum,* are commercially available.

*L. plantarum* is also known as the most frequently reported LAB species with antifungal activity [16,30,31]. In this study, the optimal culture conditions for strain 3121M0s were determined at 30 °C for 48 h based on its antiyeast activity in order to streamline the experiment (Supplemental Figure S3 and Figure 2). The antiyeast activity of 3121M0s-CFS was detected on and after 12 h of incubation at 30 °C, and was maintained at high levels for 24–72 h. Traditional fermented milk products are generally produced by incubation at ambient temperature, depending on climatic conditions, 15–45 °C, from one night to three days [25,26,27,72]. In the case of airag, incubation at 20–30 °C for 12–24 h is general [46,49,72]. These findings indicate that some antiyeast substances would be produced by *L. plantarum* in traditional fermented milk, including airag. This indication is supported by our previous study that another *L. plantarum* strain derived from airag significantly decreased the ethanol production from *S. cerevisiae* 4C cocultivated in RSM [50]. In addition, the primary antiyeast substance from strain 3121M0s was determined to be AA (Figure 7). AA production has actually been confirmed in airag in the other reports [46,73].

The antifungal effect of AA involves lowering the environmental pH beyond the growth limits of target organisms, similar to other OAs [30,31]. In addition, AA is unique in that its higher p*K*a allows a greater proportion of undissociated molecules at comparable pH levels, facilitating membrane permeation. Once inside the cell, AA dissociates, causing intracellular acidification and accumulation of anions, leading to metabolic inhibition and stronger growth suppression than other OA. In addition, AA is known to have a synergistic antimicrobial effect with LA [74,75], and this phenomenon was clearly observed also in this antiyeast study (Figure 7B,C). The antifungal effect of LA itself has not been clearly investigated, and to our knowledge, its effect alone against yeast has not been confirmed. In fact, no effects were observed in this study for LA alone or LA combined with any OA used here except AA (Figure 7A,B). The synergistic effect of LA and AA is thought to be exerted when a large amount of LA lowers the environmental pH, thereby reaching a pH range that increases the amount of undissociated AA [74,75]. In addition, it is thought that LA, together with AA, permeates the membrane, further lowering the intracellular pH and inhibiting metabolism of the target cells. Similar to LA, PLA did not show any antiyeast activity on its own, but a synergistic effect was observed with AA (Figure 7A,C). The reason that the removal of PLA had no observed effect on the activity of the mixture in Figure 7B is thought because the combination of AA and LA in the mixture already reached the maximum activity. PLA is known to disrupt membrane integrity, causing leakage of intracellular components, and to inhibit DNA replication through intercalation [76]. Based on this, the synergistic effect of AA and PLA can be explained as follows: AA lowers the pH, increasing the proportion of undissociated PLA (p*K*a 3.5–3.8), which improves membrane permeability and, as a result, facilitates the penetration of AA into the target bacterial cells [32]. However, in this research, pH was adjusted to 4.0 in most cases, so the synergistic effect between AA and the other two OAs is unlikely to be due to an increase in their undissociated forms. Conversely, the increase in antiyeast activity observed by lowering solution pH is not due to the pH falling below the growth-permissible range of the target yeast strain (*R. mucilaginosa* JCM 8115^T^), because no activity was observed in the pH-adjusted control without OAs (Figure 3). Instead, it is likely due to an increase in the undissociated forms of each OA. Additionally, OHPLA and OHBA were detected in HPLC (Figure 5 and Table 3), but due to their low concentrations, neither showed individual activity nor synergistic effects with the other OAs (Figure 7). Also, other OAs known to have antiyeast activity, such as those used as standards in HPLC, were not detected by this analytical method.

*L. plantarum* is a facultative heterofermentater known to produce relatively high levels of AA, compared to other LAB that are not obligate heterofermentaters [77]. Although the mechanism of AA production by *L. plantarum* has not been fully elucidated, it can be generally described as follows [77,78,79,80]. During the exponential phase under static cultivation in milk or MRS broth, both of which are rich in glucose (hexose), *L. plantarum* metabolizes glucose to pyruvic acid through anaerobic glycolysis and subsequently converts it almost exclusively to LA by lactate dehydrogenase. However, mainly in the early stationary phase, as glucose decreases and NADH accumulates, repression by catabolite control protein A (CcpA) is alleviated, leading to the upregulation of enzymes involved in mixed acid fermentation. Consequently, pyruvic acid is transformed into acetyl phosphate anaerobically by pyruvate formate lyase and phosphotransacetylase, or aerobically by pyruvate oxidase. Finally, AA is generated from acetyl phosphate by acetate kinase. In strain 3121M0s, although LA production was equivalent to that of the type strain, JCM 1149^T^, AA production was approximately 1.6 times higher (Table 3). This may be due to the differences in the activity of AA biosynthesis-related enzymes and/or their expression levels affected by CcpA. In addition, since ATP is generated by acetate kinase in AA production, it is predicted that the bacterial growth would improve. In fact, between the two strains, although there was no significant difference in culture pH because lactic acid production was equivalent, there was a clear difference in culture turbidity (Supplementary Figure S5). Considering the future use of strain 3121M0s, further optimization of cultivation conditions is required to achieve higher AA production, as it is known that AA production increases by oxygenating as well as restricting glucose [77,78,79,80].

Besides, strain 3121M0s produced approximately 1.4 times more PLA than the type strain (Table 3). This is also thought to contribute to the higher antiyeast activity of 3121M0s (Table 4). The PLA production furthermore increased approximately 1.3-fold (Table 5) when 0.4% Phe was added to the medium based on previous reports [62]. Similarly, the addition of 0.4% Tyr increased OHPLA production by 4.4-fold. However, no increase in activity was observed with the addition. Phe and Tyr are oxidatively deaminated to phenylpyruvic acid and 4-hydroxyphenylpyrubic acid by an aromatic aminotransferase, and subsequently they are converted to PLA and OHPLA by a hydroxyl acid dehydrogenase, respectively [81,82,83]. These reactions, which are NADH-dependent, explain the increased PLA and OHPLA production by the addition of Phe and Tyr, and also contribute to the elimination of the accumulated NADH. Interestingly, the Phe addition led to an increase in PLA alongside a significant decrease in OHPLA production, while the Tyr addition resulted in an increase in OHPLA production alongside a significant decrease in PLA production. Similar reports have been published in the past, and this is thought to be due to the use of the same enzymes in the production of both OAs [62]. Despite the increased production of PLA and OHPLA, the activity remained unchanged, which is thought to be due to their effect being masked by the already high effect of the combination of AA and LA.

In this study, the CFS of strain 3121M0s was extracted with ethyl acetate, and the organic layer was used for qualitative and quantitative analyses of OA. A slight difference in activity was observed between 10× CFS and the corresponding OA mixture (Table 4), and heat (at 121 °C for 15 min) and protease treatments led to a slight decrease in activity (Figure 4). These results suggest the presence of proteinaceous antiyeast substances, similar to those previously reported [34,35,36,37,38,84]. Since these substances might be present in another (aqueous) layer on the ethyl acetate separation, current efforts are focused on identifying antifungal substances in the layer.

In conclusion, this study is the first to comprehensively characterize the antiyeast activity and synergistic effect of OAs produced by *L. plantarum* 3121M0s isolated from traditional Mongolian fermented milk, airag. The detailed analysis of both individual and combined effects of LA, AA, and PLA provided clear evidence of their synergistic antiyeast activity, which had previously only been vaguely described. Given the large genome and complex metabolism of *L. plantarum* [85], further studies are needed to fully elucidate its antifungal capabilities. As *L. plantarum* 3121M0s is capable of milk fermentation, it holds promise as a biopreservative starter for fermented dairy foods produced in open environments without final sterilization, where yeast contamination is a significant concern.

## Figures and Tables

**Table 1:** Antiyeast activity of 236 strains and tenfold concentrates (pH 4.0) of the cell-free culture supernatants of the selected four strains of lactic acid bacteria against three strains of food-related yeasts using the overlay and agar–well diffusion methods, respectively.

Strain	Overlay Assay * Against				Agar–Well Diffusion Assay ** Against		
	*Rhodotorula* *mucilaginosa*	*Candida* *parapsilosis*	*Saccharomyces* *cerevisiae*		*Rhodotorula* *mucilaginosa*	*Candida* *parapsilosis*	*Saccharomyces* *cerevisiae*
	JCM 8115^T^	JCM 1612^T^	4C		JCM 8115^T^	JCM 1612^T^	4C
3121M0s	+++	++	-		10.63 ± 0.45	-	-
3272B0	+++	++	-		-	-	-
3273B0	+++	++	-		-	-	-
3461M0	+++	++	-		-	-	-
123	+++	±	-		N.D.	N.D.	N.D.
IB3781	+++	±	-		N.D.	N.D.	N.D.
NMCB2521	+++	±	-		N.D.	N.D.	N.D.
NMCM2521	+++	±	-		N.D.	N.D.	N.D.
3070	+++	-	-		N.D.	N.D.	N.D.
301102S	+++	-	-		N.D.	N.D.	N.D.
425	+++	-	-		N.D.	N.D.	N.D.
IM376	++	+	-		N.D.	N.D.	N.D.
IB374	+	+	-		N.D.	N.D.	N.D.
JCM 1120^T^	-	+	-		N.D.	N.D.	N.D.
2253RB0	-	+	-		N.D.	N.D.	N.D.
5890	-	-	±		N.D.	N.D.	N.D.
50 strains	++	-	-		N.D.	N.D.	N.D.
77 strains	+	-	-		N.D.	N.D.	N.D.
93 strains	-	-	-		N.D.	N.D.	N.D.

* -, no activity; ±, growth delay; +, inhibitory diameter (ID) < 11 mm; ++, ID 11–15 mm; and +++, ID > 15 mm. ** The value represents ID length (mean ± S.D.; mm). -, no activity; and N.D., No data.

**Table 2:** Antiyeast activity of strain 3121M0s against eight strains of food spoilage and fermentation yeasts using the overlay and agar–well diffusion methods.

Assay	*Debaryomyces* *hansenii*	*Hanseniaspora* *uvarum*	*Issatchenkia* *orientalis*	*Kluyveromyces* *marxianus*	*Pichia* *kudriavzevii*	*Saccharomyces* *cerevisiae*	*Wickerhamomyces* *anomalus*	*Zygosaccharomyces* *rouxii*
	JCM 1990^T^	JCM 2184^T^	JCM 1710^T^	JCM 1617^T^	JCM 3536^T^	JCM 7255^T^	JCM 3585^T^	JCM 2325^T^
Overlay *	++	+	-	+	-	+	-	+
Agar–well diffusion **	10.83 ± 0.24	8.67 ± 0.24	-	7.00 ± 0.00	-	-	-	7.33 ± 0.24

* -, no activity; +, inhibitory diameter (ID) < 11 mm; ++, and ID 11–15 mm. ** Tenfold concentrate (pH 4.0) of the cell-free culture supernatant of strain 3121M0s was used as a sample. The value represents ID length (mean ± S.D.; mm). -, no activity.

**Table 3:** Contents of organic acids in the cell-free culture supernatants (CFS) of *Lactiplantibacillus plantarum* 3121M0s and JCM 1149^T^, calculated based on the area of each HPLC peak.

Ingredient	Concentration (mg/L)	
	3121M0s-CFS	JCM 1149^T^-CFS
DL-Lactic acid	16,053.0	16,244.4
(D-form:L-form)	(10,981.0:5072.0)	(11,571.9:4672.5)
Acetic acid	1437.9	902.2
4-Hydroxyphenyllactic acid	28.7	3.0
4-Hydroxybenzoic acid	26.9	34.4
3-Phenyllactic acid	99.1	69.2

**Table 4:** Antiyeast activity of the tenfold cell-free culture supernatants (pH 4.0) and organic acids mixtures (pH 4.0) of *Lactiplantibacillus plantarum* 3121M0s and JCM 1149^T^ against *Rhodotorula mucilaginosa* JCM 8115^T^.

Solution	Antiyeast Activity (mm; Mean ± S.D.) *		
	3121M0s	JCM 1149^T^	MRS Broth
Tenfold culture supernatant **	10.63 ± 0.45 ^a^	7.00 ± 0.00 ^c^	- ^d^
Tenfold organic acids mixture ***	10.00 ± 0.00 ^b^	6.83 ± 0.24 ^c^	N.D.

* -, no activity; and N.D., no data. Different lowercase letters denote a significant difference (*p* < 0.05). ** The cell-free culture supernatants were obtained after cultivation of the two strains in MRS broth at 30 °C for 48 h. *** The mixtures of organic acids were prepared on the basis of the values shown in Table 3.

**Table 5:** Effects of adding L-tyrosine and L-phenylalanine to MRS broth on the production of 4-hydroxyphenyllactic acid and 3-phenyllactic acid, and on the antiyeast activity against *Rhodotorula mucilaginosa* JCM 8115^T^ in the cell-free supernatant (CFS) of strain 3121M0s.

Additive to MRS Broth	Concentration (mg/L) in 3121M0s-CFS		Antiyeast Activity (mm; Mean ± S.D.) of 10-Fold 3121M0s-CFS (pH 4.0) *
	4-Hydroxyphenyllactic Acid	3-Phenyllactic Acid	
No added	28.7	99.1	10.63 ± 0.45 ^a^
L-Tyrosine	125.4	Trace	11.00 ± 0.00 ^a^
L-Phenylalanine	Trace	126.5	11.00 ± 0.00 ^a^
L-Tyrosin and L-phenylalanine	84.9	88.9	11.00 ± 0.00 ^a^

* Antiyeast activity assay was performed against *Rhodotorula mucilaginosa* JCM 8115^T^. Different lowercase letters denote a significant difference (*p* < 0.05).

## Data Availability

Genetic data from this study have been deposited to DDBJ under accession number LC884927. The original contributions presented in the study are included in the article. Further inquiries can be directed to the corresponding author.

## Supplementary Materials

The following supporting information can be downloaded at https://www.mdpi.com/article/10.3390/microorganisms13092017/s1, Figure S1: Phylogenetic tree based on 16S rRNA gene sequences showing the relationship of strain 3121M0s with the type strains of all species of the genus *Lactiplantibacillus*; Figure S2: Electrophoresis profile of the *recA* multiplex PCR amplicons; Figure S3: Changes in culture turbidity of strain 3121M0s cultivated in MRS broth at 25, 30, and 37 °C (light blue, red, and yellow green, respectively) for 72 h; Figure S4: FAB-MS (a,b) and EI-MS (c–j) spectra of standard chemicals (a,c,e,g,i) and fractions of HPLC peaks (b,d,f,h,j) described in Figure 5; Figure S5: Changes in culture pH (broken lines) and turbidity (solid lines) of *Lactiplantibacillus plantarum* 3121M0s (light blue) and JCM 1149^T^ (red) cultivated in MRS broth at 30 °C for 72 h.

## Abbreviations

The following abbreviations are used in this manuscript:

LAB	Lactic acid bacteria
AA	Acetic acid
PLA	3-Phenyllactic acid
MRS	De Man, Rogosa, and Sharp
YM	Yeast and Mold
RSM	Reconstituted skim milk
S.D.	Standard deviation
CFU	Colony forming unit
CFS	Cell-free culture supernatant
10× CFS	Tenfold concentrated cell-free culture supernatant
OAs	Organic acids
rRNA	Ribosomal ribonucleic acid
PCR	Polymerase chain reaction
DNA	Deoxyribonucleic acid
BLAST	Basic Local Alignment Search Tool
NCBI	National Center for Biotechnology Information
DDBJ	DNA Data Bank Japan
OD	Optical density
HPLC	High performance liquid chromatography
OHBA	4-Hydroxybenzoic acid
OHPLA	4-hydroxyphenyllactic acid
LA	Lactic acid
MS	Mass spectrometry
FAB-MS	Fast atom bombardment mass spectrometry
EI-MS	Electron ionization mass spectrometry
Tyr	Tyrosine
Phe	Phenylalanine
ANOVA	Analysis of variance
NADH	Reduced nicotinamide adenine dinucleotide
CcpA	catabolite control protein A
ATP	Adenosine triphosphate

## References

[1] Snyder A.B., Worobo R.W. (2018). Fungal Spoilage in Food Processing. J. Food Prot..

[2] Gallo M., Ferrara L., Calogero A., Montesano D., Naviglio D. (2020). Relationships between food and diseases: What to know to ensure food safety. Food Res. Int..

[3] Hymery N., Vasseur V., Coton M., Mounier J., Jany J.-L., Barbier G., Coton E. (2014). Filamentous Fungi and Mycotoxins in Cheese: A Review. Compr. Rev. Food Sci. Food Saf..

[4] Khan R., Anwar F., Mohamad Ghazali F. (2024). A comprehensive review of mycotoxins: Toxicology, detection, and effective mitigation approaches. Heliyon.

[5] Mafe A.N., Büsselberg D. (2024). Mycotoxins in Food: Cancer Risks and Strategies for Control. Foods.

[6] Bintsis T. (2021). Yeasts in different types of cheese. AIMS Microbiol..

[7] Libkind D., Peris D., Cubillos F.A., Steenwyk J.L., Opulente D.A., Langdon Q.K., Rokas A., Hittinger C.T. (2020). Into the wild: New yeast genomes from natural environments and new tools for their analysis. FEMS Yeast Res..

[8] Kovács M., Pomázi A., Taczman-Brückner A., Kiskó G., Dobó V., Kocsis T., Mohácsi-Farkas C., Belák Á. (2025). Detection and Identification of Food-Borne Yeasts: An Overview of the Relevant Methods and Their Evolution. Microorganisms.

[9] Fleet G.H. (1990). Yeasts in Dairy Products. J. Appl. Bacteriol..

[10] Viljoen B.C., Greyling T. (1995). Yeasts associated with Cheddar and Gouda making. Int. J. Food Microbiol..

[11] Fleet G.H. (2007). Yeasts in foods and beverages: Impact on product quality and safety. Curr. Opin. Biotechnol..

[12] Sagdic O., Ozturk I., Bayram O., Kesmen Z., Yilmaz M.T. (2010). Characterization of Butter Spoiling Yeasts and Their Inhibition by Some Spices. J. Food Sci..

[13] Melville P.A., Benites N.R., Ruz-Peres M., Yokoya E. (2011). Proteinase and phospholipase activities and development at different temperatures of yeasts isolated from bovine milk. J. Dairy Res..

[14] Moubasher A.H., Abdel-Sater M.A., Soliman Z.S.M. (2018). Yeasts and filamentous fungi associated with some dairy products in Egypt. J. Mycol. Med..

[15] Hernández A., Pérez-Nevado F., Ruiz-Moyano S., Serradilla M.J., Villalobos M.C., Martín A., Córdoba M.G. (2018). Spoilage yeasts: What are the sources of contamination of foods and beverages?. Int. J. Food Microbiol..

[16] Riešutė R., Šalomskienė J., Saez Moreno D., Gustienė S. (2021). Effect of yeasts on food quality and safety and possibilities of their inhibition. Trends Food Sci. Technol..

[17] Geronikou A., Srimahaeak T., Rantsiou K., Triantafillidis G., Larsen N., Jespersen L. (2020). Occurrence of Yeasts in White-Brined Cheeses: Methodologies for Identification, Spoilage Potential and Good Manufacturing Practices. Front. Microbiol..

[18] Geronikou A., Larsen N., Lillevang S.K., Jespersen L. (2022). Occurrence and Identification of Yeasts in Production of White-Brined Cheese. Microorganisms.

[19] Geronikou A., Larsen N., Lillevang S.K., Jespersen L. (2023). Diversity and succession of contaminating yeasts in white-brined cheese during cold storage. Food Microbiol..

[20] de Melo Pereira G.V., Maske B.L., de Carvalho Neto D.P., Karp S.G., De Dea Lindner J., Martin J.G.P., Hosken B.O., Soccol C.R. (2022). What Is Candida Doing in My Food? A Review and Safety Alert on Its Use as Starter Cultures in Fermented Foods. Microorganisms.

[21] Wirth F., Goldani L.Z. (2012). Epidemiology of *Rhodotorula*: An emerging pathogen. Interdiscip. Perspect. Infect. Dis..

[22] Enache-Angoulvant A., Hennequin C. (2005). Invasive *Saccharomyces* infection: A comprehensive review. Clin. Infect. Dis..

[23] Shen D., He X., Weng P., Liu Y., Wu Z. (2022). A review of yeast: High cell-density culture, molecular mechanisms of stress response and tolerance during fermentation. FEMS Yeast Res..

[24] Rohm H., Lechner F., Lehner M. (1990). Microflora of Austrian natural-set yogurt. J. Food Prot..

[25] Ayoub M.-J., Bechara P., Habchi M., Hosry R., Akl M., Haj Hassan S., Abi Nakhoul P. (2022). Raw goat’s milk fermented Anbaris from Lebanon: Insights into the microbial dynamics and chemical changes occurring during artisanal production, with a focus on yeasts. J. Dairy Res..

[26] Karssa T.H., Kussaga J.B., Semedo-Lemsaddek T., Mugula J.K. (2024). Insights on the microbiology of Ethiopian fermented milk products: A review. Food Sci. Nutr..

[27] Chandran A., Beena A.K. (2024). Impact of climate and traditional practice on quality of homemade dahi. J. Dairy Res..

[28] Garnier L., Valence F., Pawtowski A., Auhustsinava-Galerne L., Frotté N., Baroncelli R., Deniel F., Coton E., Mounier J. (2017). Diversity of spoilage fungi associated with various French dairy products. Int. J. Food Microbiol..

[29] Bento de Carvalho T., Nunes Silva B., Tomé E., Teixeira P. (2024). Preventing fungal spoilage from raw materials to final product: Innovative preservation techniques for fruit fillings. Foods.

[30] Crowley S., Mahony J., van Sinderen D. (2013). Current perspectives on antifungal lactic acid bacteria as natural bio-preservatives. Trends Food Sci. Technol..

[31] Abouloifa H., Hasnaoui I., Rokni Y., Bellaouchi R., Ghabbour N., Karboune S., Brasca M., Abousalham A., Jaouadi B., Saalaoui E. (2022). Chapter Two—Antifungal activity of lactic acid bacteria and their application in food biopreservation. Adv. Appl. Microbiol..

[32] Nasrollahzadeh A., Mokhtari S., Khomeiri M., Saris P.E.J. (2022). Antifungal preservation of food by lactic acid bacteria. Foods.

[33] Liang N., Zhao Z., Curtis J.M., Gänzle M.G. (2022). Antifungal cultures and metabolites of lactic acid bacteria for use in dairy fermentations. Int. J. Food Microbiol..

[34] Ström K., Sjögren J., Broberg A., Schnürer J. (2002). *Lactobacillus plantarum* MiLAB 393 produces the antifungal cyclic dipeptides cyclo(L-Phe-L-Pro) and cyclo(L-Phe-trans-4-OH-L-Pro) and 3-phenyllactic acid. Appl. Environ. Microbiol..

[35] Yépez A., Luz C., Meca G., Vignolo G., Mañes J., Aznar R. (2017). Biopreservation potential of lactic acid bacteria from Andean fermented food of vegetal origin. Food Control.

[36] Sjögren J., Magnusson J., Broberg A., Schnürer J., Kenne L. (2003). Antifungal 3-hydroxy fatty acids from *Lactobacillus plantarum* MiLAB 14. Appl. Environ. Microbiol..

[37] Vimont A., Fernandez B., Ahmed G., Pilote Fortin H., Fliss I. (2019). Quantitative antifungal activity of reuterin against food isolates of yeasts and moulds and its potential application in yogurt. Int. J. Food Microbiol..

[38] Zheng X., Nie W., Xu J., Zhang H., Liang X., Chen Z. (2022). Characterization of antifungal cyclic dipeptides of *Lacticaseibacillus paracasei* ZX1231 and active packaging film prepared with its cell-free supernatant and bacterial nanocellulose. Food Res. Int..

[39] Belguesmia Y., Choiset Y., Rabesona H., Baudy-Floc’h M., Le Blay G., Haertlé T., Chobert J.-M. (2013). Antifungal properties of durancins isolated from *Enterococcus durans* A5-11 and of its synthetic fragments. Lett. Appl. Microbiol..

[40] Okkers D.J., Dicks L.M., Silvester M., Joubert J.J., Odendaal H.J. (1999). Characterization of pentocin TV35b, a bacteriocin-like peptide isolated from *Lactobacillus pentosus* with a fungistatic effect on *Candida albicans*. J. Appl. Microbiol..

[41] Yang E., Fan L., Jiang Y., Doucette C., Fillmore S. (2012). Antimicrobial activity of bacteriocin-producing lactic acid bacteria isolated from cheeses and yogurts. AMB Express.

[42] Magnusson J., Schnürer J. (2001). *Lactobacillus coryniformis* subsp. coryniformis strain Si3 produces a broad-spectrum proteinaceous antifungal compound. Appl. Environ. Microbiol..

[43] Watanabe K., Fujimoto J., Sasamoto M., Dugersuren J., Tumursuh T., Demberel S. (2008). Diversity of lactic acid bacteria and yeasts in Airag and Tarag, traditional fermented milk products of Mongolia. World J. Microbiol. Biotechnol..

[44] Sun Z.H., Liu W.J., Zhang J.C., Yu J., Gao W., Jiri M., Menghe B., Sun T.S., Zhang H.P. (2010). Identification and characterization of the dominant lactic acid bacteria isolated from traditional fermented milk in Mongolia. Folia Microbiol..

[45] Takeda S., Yamasaki K., Takeshita M., Kikuchi Y., Tsend-Ayush C., Dashnyam B., Ahhmed A.M., Kawahara S., Muguruma M. (2011). The investigation of probiotic potential of lactic acid bacteria isolated from traditional Mongolian dairy products. Anim. Sci. J..

[46] Choi S.-H. (2016). Characterization of airag collected in Ulaanbaatar, Mongolia with emphasis on isolated lactic acid bacteria. J. Anim. Sci. Technol..

[47] Oyundelger G., Sumisa F., Batjargal B., Yoshida T. (2016). Isolation and identification of new lactic acid bacteria with potent biological activity and yeasts in Airag, a traditional Mongolian fermented beverage. Food Sci. Technol. Res..

[48] Oki K., Dugersuren J., Demberel S., Watanabe K. (2014). Pyrosequencing analysis of the microbial diversity of airag, khoormog and tarag, traditional fermented dairy products of Mongolia. Biosci. Microbiota Food Health.

[49] Shinoda A., Koga Y., Tsuchiya R., Tserenpurev B.-O., Battsetseg B., Morinaga Y., Nakayama J. (2025). Impact of container type on the microbiome of airag, a Mongolian fermented mare’s milk. Biosci. Microbiota Food Health.

[50] Sudun, Wulijideligen, Arakawa K., Miyamoto M., Miyamoto T. (2013). Interaction between lactic acid bacteria and yeasts in airag, an alcoholic fermented milk. Anim. Sci. J..

[51] Miyamoto M., Ueno H.M., Watanabe M., Tatsuma Y., Seto Y., Miyamoto T., Nakajima H. (2015). Distinctive proteolytic activity of cell envelope proteinase of *Lactobacillus helveticus* isolated from airag, a traditional Mongolian fermented mare’s milk. Int. J. Food Microbiol..

[52] Ganzorig B., Zayabaatar E., Pham M.T., Marito S., Huang C.-M., Lee Y.-H. (2023). *Lactobacillus plantarum* Generate Eelectricity through Flavin Mononucleotide-Mediated Extracellular Electron Transfer to Upregulate Epithelial Type I Collagen Expression and Thereby Promote Microbial Adhesion to Intestine. Biomedicines.

[53] Galindev U., Erdenebold U., Batnasan G., Ganzorig O., Batdorj B. (2024). Anti-obesity effects of potential probiotic *Lactobacillus* strains isolated from Mongolian fermented dairy products in high-fat diet-induced obese rodent model. Braz. J. Microbiol..

[54] Batdorj B., Dalgalarrondo M., Choiset Y., Pedroche J., Métro F., Prévost H., Chobert J.-M., Haertlé T. (2006). Purification and characterization of two bacteriocins produced by lactic acid bacteria isolated from Mongolian airag. J. Appl. Microbiol..

[55] Wulijideligen, Asahina T., Hara K., Arakawa K., Nakano H., Miyamoto T. (2012). Production of bacteriocin by *Leuconostoc mesenteroides* 406 isolated from Mongolian fermented mare’s milk, airag. Anim. Sci. J..

[56] Arakawa K., Yoshida S., Aikawa H., Hano C., Bolormaa T., Burenjargal S., Miyamoto T. (2016). Production of a bacteriocin-like inhibitory substance by *Leuconostoc mesenteroides* subsp. dextranicum 213M0 isolated from Mongolian fermented mare milk, airag. Anim. Sci. J..

[57] Hasiqimuge, Hano C., Arakawa K., Yoshida S., Zhao J., Toh H., Morita H., Miyamoto T. (2024). A novel C-terminal truncated bacteriocin found by comparison between *Leuconostoc mesenteroides* 406 and 213M0 isolated from Mongolian traditional fermented milk, airag. Microorganisms.

[58] Arakawa K., Kawai Y., Iioka H., Tanioka M., Nishimura J., Kitazawa H., Koga M., Saito T., Itoh T. (2008). Microbial Community Analysis of Food-Spoilage Bacteria in Commercial Custard Creams Using Culture-Dependent and Independent Methods. J. Dairy Sci..

[59] Klaenhammer T.R. (1993). Genetics of Bacteriocins Produced by Lactic Acid Bacteria. FEMS Microbiol. Rev..

[60] Castresana J. (2000). Selection of conserved blocks from multiple alignments for their use in phylogenetic analysis. Mol. Biol. Evol..

[61] Torriani S., Felis G.E., Dellaglio F. (2001). Differentiation of *Lactobacillus plantarum*, *L. pentosus*, and *L. paraplantarum* by *recA* Gene Sequence Analysis and Multiplex PCR Assay with *recA* Gene-Derived Primers. Appl. Environ. Microbiol..

[62] Valerio F., Lavermicocca P., Pascale M., Visconti A. (2004). Production of Phenyllactic Acid by Lactic Acid Bacteria: An Approach to the Selection of Strains Contributing to Food Quality and Preservation. FEMS Microbiol. Lett..

[63] Crowley S., Mahony J., van Sinderen D. (2012). Comparative analysis of two antifungal *Lactobacillus plantarum* isolates and their application as bioprotectants in refrigerated foods. J. Appl. Microbiol..

[64] Leyva Salas M., Thierry A., Lemaître M., Garric G., Harel-Oger M., Chatel M., Lê S., Mounier J., Valence F., Coton E. (2018). Antifungal Activity of Lactic Acid Bacteria Combinations in Dairy Mimicking Models and Their Potential as Bioprotective Cultures in Pilot Scale Applications. Front. Microbiol..

[65] Afzali S., Edalatian Dovom M.R., Habibi Najafi M.B., Mazaheri Tehrani M. (2020). Determination of the anti-yeast activity of *Lactobacillus* spp. isolated from traditional Iranian cheeses in vitro and in yogurt drink (Doogh). Sci. Rep..

[66] Makki G.M., Kozak S.M., Jencarelli K.G., Alcaine S.D. (2020). Evaluation of the efficacy of commercial protective cultures against mold and yeast in queso fresco. J. Dairy Sci..

[67] Makki G.M., Kozak S.M., Jencarelli K.G., Alcaine S.D. (2021). Evaluation of the efficacy of commercial protective cultures to inhibit mold and yeast in cottage cheese. J. Dairy Sci..

[68] de Vries M.C., Vaughan E.E., Kleerebezem M., de Vos W.M. (2006). *Lactobacillus plantarum*—Survival, functional and potential probiotic properties in the human intestinal tract. Int. Dairy J..

[69] Siezen R.J., van Hylckama Vlieg J.E.T. (2011). Genomic diversity and versatility of *Lactobacillus plantarum*, a natural metabolic engineer. Microb. Cell Fact..

[70] Fidanza M., Panigrahi P., Kollmann T.R. (2021). *Lactiplantibacillus plantarum*—Nomad and Ideal Probiotic. Front. Microbiol..

[71] Yilmaz B., Bangar S.P., Echegaray N., Suri S., Tomasevic I., Lorenzo J.M., Melekoglu E., Rocha J.M., Ozogul F. (2022). The Impacts of *Lactiplantibacillus plantarum* on the Functional Properties of Fermented Foods: A Review of Current Knowledge. Microorganisms.

[72] Bintsis T., Papademas P. (2022). The Evolution of Fermented Milks, from Artisanal to Industrial Products: A Critical Review. Fermentation.

[73] Ganzorig O., Batdorj B., Ishii S. (2025). Characterization of volatile compound profile in Mongolian traditional fermented mare’s milk, as Airag. Anim. Sci. J..

[74] Mbandi E., Shelef L.A. (2001). Enhanced inhibition of *Listeria monocytogenes* and *Salmonella enteritidis* in meat by combinations of sodium lactate and diacetate. J. Food Prot..

[75] Ji Q.-Y., Wang W., Yan H., Qu H., Liu Y., Qian Y., Gu R. (2023). The Effect of Different Organic Acids and Their Combination on the Cell Barrier and Biofilm of *Escherichia coli*. Foods.

[76] Zhang C., Wang Y., Guo M., Kong Y., Fan X., Sun S., Du C., Gong H. (2025). Antifungal mechanisms of phenyllactic acid against *Mucor racemosus*: Insights from spore growth suppression and proteomic analysis. Food Chem..

[77] Lorquet F., Goffin P., Muscariello L., Baudry J.-B., Ladero V., Sacco M., Kleerebezem M., Hols P. (2004). Characterization and functional analysis of the *poxB* gene, which encodes pyruvate oxidase in *Lactobacillus plantarum*. J. Bacteriol..

[78] Goffin P., Lorquet F., Kleerebezem M., Hols P. (2004). Major Role of NAD-Dependent Lactate Dehydrogenases in Aerobic Lactate Utilization in *Lactobacillus plantarum* during Early Stationary Phase. J. Bacteriol..

[79] Zotta T., Parente E., Ricciardi A. (2017). Aerobic Metabolism in the Genus *Lactobacillus*: Impact on Stress Response and Potential Applications in the Food Industry. J. Appl. Microbiol..

[80] Lu Y., Song S., Tian H., Yu H., Zhao J., Chen C. (2018). Functional Analysis of the Role of CcpA in *Lactobacillus plantarum* Grown on Fructooligosaccharides or Glucose: A Transcriptomic Perspective. Microb. Cell Fact..

[81] Dallagnol A.M., Catalán C.A.N., Mercado M.I., Font de Valdez G., Rollán G.C. (2011). Effect of biosynthetic intermediates and citrate on the phenyllactic and hydroxyphenyllactic acids production by *Lactobacillus plantarum* CRL 778. J. Appl. Microbiol..

[82] Li M., Meng X., Sun Z., Zhu C., Ji H. (2019). Effects of NADH availability on 3-phenyllactic acid production by *Lactobacillus plantarum* expressing formate dehydrogenase. Curr. Microbiol..

[83] Dao Y., Zhang K., Lu X., Lu Z., Liu C., Liu M., Luo Y. (2019). Role of glucose and 2-oxoglutarate/malate translocator (OMT1) in the production of phenyllactic acid and *p*-hydroxyphenyllactic acid, two food-borne pathogen inhibitors. J. Agric. Food Chem..

[84] Abouloifa H., Rokni Y., Hasnaoui I., Bellaouchi R., Gaamouche S., Ghabbour N., Karboune S., Ben Salah R., Brasca M., D’hallewin G. (2022). Characterization of antimicrobial compounds obtained from the potential probiotic *Lactiplantibacillus plantarum* S61 and their application as a biopreservative agent. Braz. J. Microbiol..

[85] Plumed-Ferrer C., Koistinen K.M., Tolonen T.L., Lehesranta S.J., Kärenlampi S.O., Mäkimattila E., Joutsjoki V., Virtanen V., von Wright A. (2008). Comparative study of sugar fermentation and protein expression patterns of two *Lactobacillus plantarum* strains grown in three different media. Appl. Environ. Microbiol..

