# Supplementary material for: Identification and Characterization of Antiyeast Organic Acids Produced by Lactiplantibacillus plantarum 3121M0s Derived from Mongolian Traditional Fermented Milk, Airag
Source: Microorganisms. 2025 Aug 29;13(9):2017. doi: 10.3390/microorganisms13092017 (PMC12472905; doi:10.3390/microorganisms13092017)
Supplement: Supplementary file 1 [file microorganisms-13-02017-s001.zip › microorganisms-3768529-supplementary.pdf]

Identification and Characterization of Antiyeast Organic Acids Produced by *Lactiplantibacillus plantarum* 3121M0s Derived from Mongolian Traditional Fermented Milk, Airag

Md. Bakhtiar Lijon[1], Yuko Matsu-ura[1], Takumi Ukita[1], Kensuke Arakawa[1,*] and Taku Miyamoto[1,2,3,4]

[1] Graduate School of Environmental and Life Science, Okayama University, Okayama 7008530, Japan

[2] Faculty of Food Culture, Kurashiki Sakuyo University, Okayama 7100292, Japan

[3] Microbial Fermentation Research Center, Minori Co., Ltd., Okayama 7011221, Japan

[4] Functional Food Creation Research Institute Co., Ltd., Okayama 7161241, Japan

[*] Correspondence: karakawa@okayama-u.ac.jp

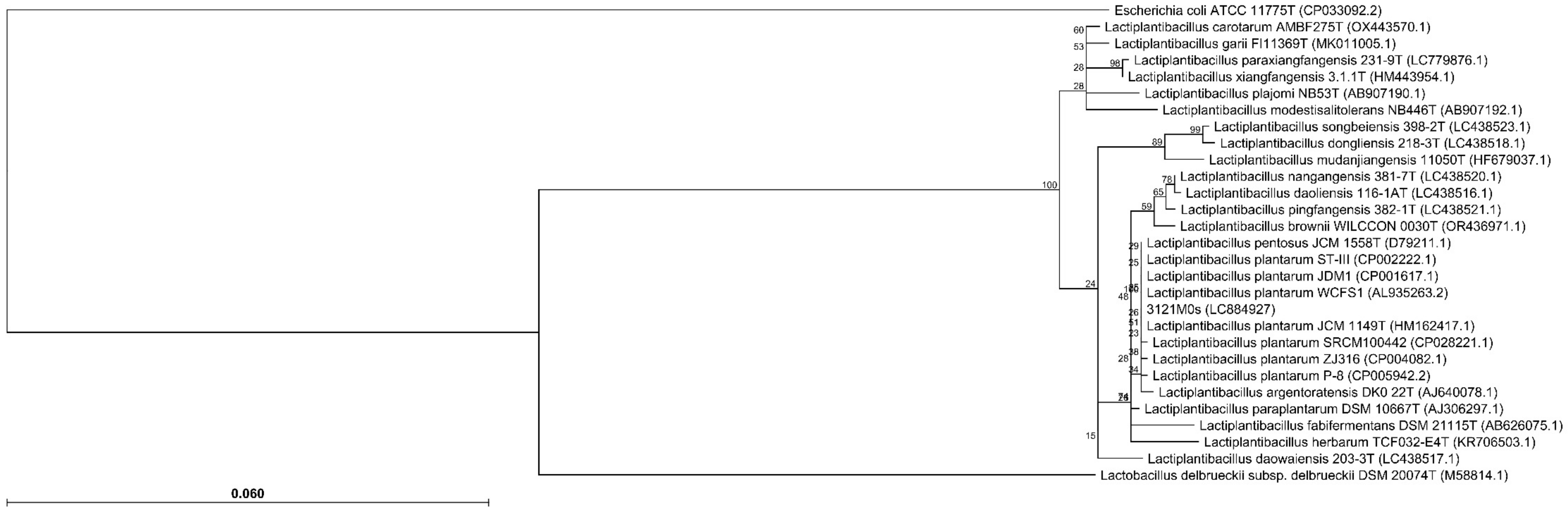

Supplementary Figure S1. Phylogenetic tree based on 16S rRNA gene sequences showing the relationship of strain 3121M0s with the type strains of all species of the genus *Lactiplantibacillus*. The type strains of *Lactobacillus delbrueckii* subsp. *bulgaricus* and *Escherichia coli* were used as outgroups. The tree was constructed using the maximum-likelihood method with the Jukes-Cantor model for a comparison of approximately 1360 bp after trimming to align the lengths. Bootstrap percentages after 500 replications are shown. The scale bar indicates 6% substitutions per nucleotide position.

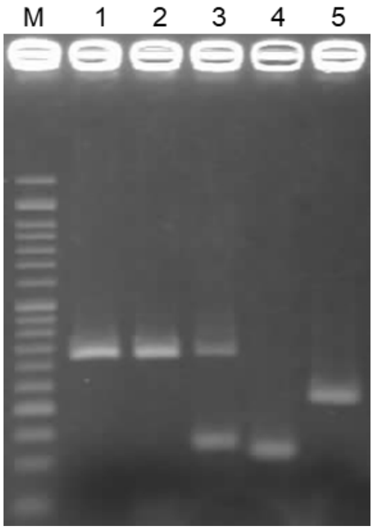

Supplementary Figure S2. Electrophoresis profile of the *recA* multiplex PCR amplicons. Lane M indicates 50-bp DNA ladder (FastGene MWD50, Nippon Genetics Co, Ltd., Tokyo, Japan). Lanes 1-5 show strain 3121M0s, *Lactiplantibacillus plantarum* JCM 1149^T, *Lactiplantibacillus argentoratensis* JCM 16169^T, *Lactiplantibacillus paraplantarum* JCM 12533^T, and *Lactiplantibacillus pentosus* JCM 1558^T, respectively.

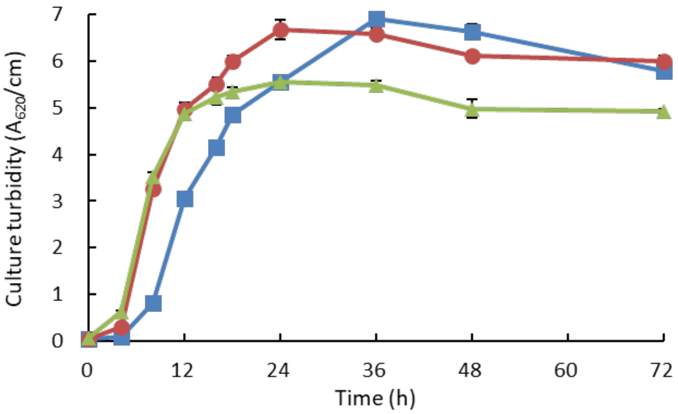

Supplementary Figure S3. Changes in culture turbidity of strain 3121M0s cultivated in MRS broth at 25, 30, and 37°C (light blue, red, and yellow green, respectively) for 72 h.

(a)

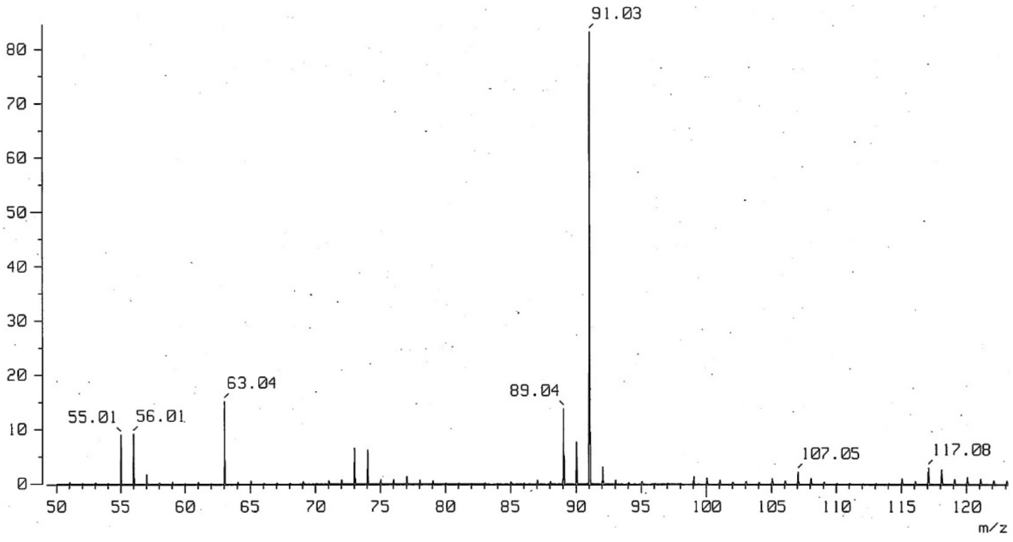

(b)

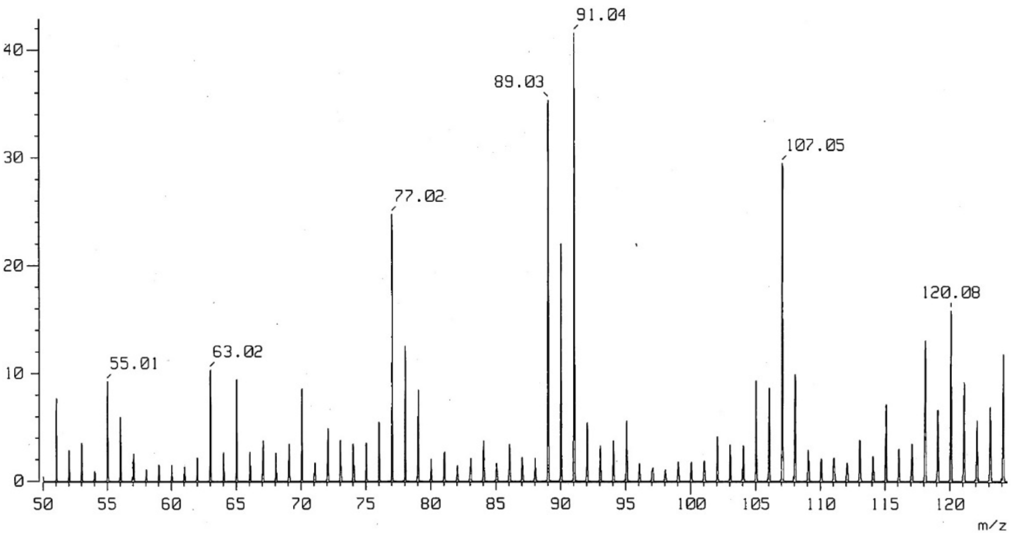

Supplementary Figure S4. FAB-MS (a, b) and EI-MS (c-j) spectra of standard chemicals (a, c, e, g, i) and fractions of HPLC peaks (b, d, f, h, j) described in Figure 5. Standards: (a) DL-Lactic acid (m/z = 91.03, $[M + H]^+$); (c) acetic acid (m/z = 60, $[M]^+$); (e) 4-Hydroxyphenyllactic acid (m/z = 182, $[M]^+$); (g) 4-Hydroxybenzoic acid (m/z = 138, $[M]^+$); (i) 3-Phenyllactic acid (m/z = 166, $[M]^+$). Samples: (b) peak A (m/z = 91.04, $[M + H]^+$); (d) peak B (m/z = 60, $[M]^+$); (f) peak C (m/z = 182, $[M]^+$); (h) peak D (m/z = 138, $[M]^+$); and (j) peak E (m/z = 166, $[M]^+$).

(c)

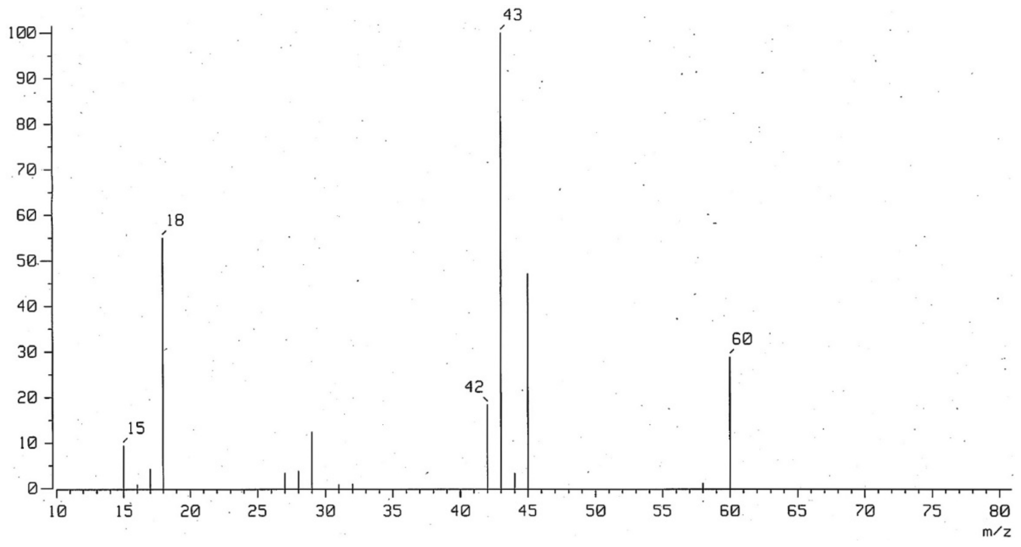

(d)

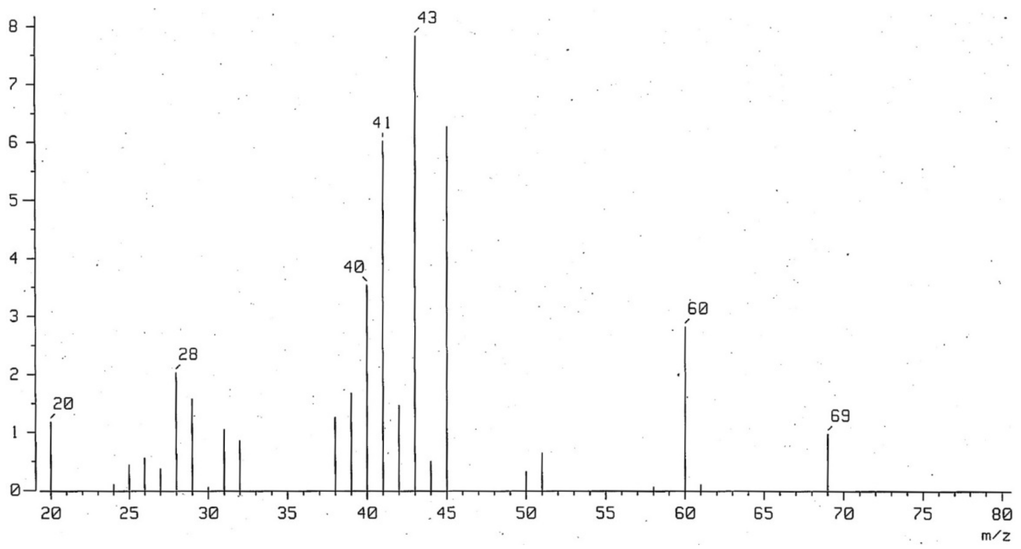

Supplementary Figure S4. FAB-MS (a, b) and EI-MS (c-j) spectra of standard chemicals (a, c, e, g, i) and fractions of HPLC peaks (b, d, f, h, j) described in Figure 5. Standards: (a) DL-Lactic acid (m/z = 91.03, $[M + H]^+$); (c) acetic acid (m/z = 60, $[M]^+$); (e) 4-Hydroxyphenyllactic acid (m/z = 182, $[M]^+$); (g) 4-Hydroxybenzoic acid (m/z = 138, $[M]^+$); (i) 3-Phenyllactic acid (m/z = 166, $[M]^+$). Samples: (b) peak A (m/z = 91.04, $[M + H]^+$); (d) peak B (m/z = 60, $[M]^+$); (f) peak C (m/z = 182, $[M]^+$); (h) peak D (m/z = 138, $[M]^+$); and (j) peak E (m/z = 166, $[M]^+$).

(e)

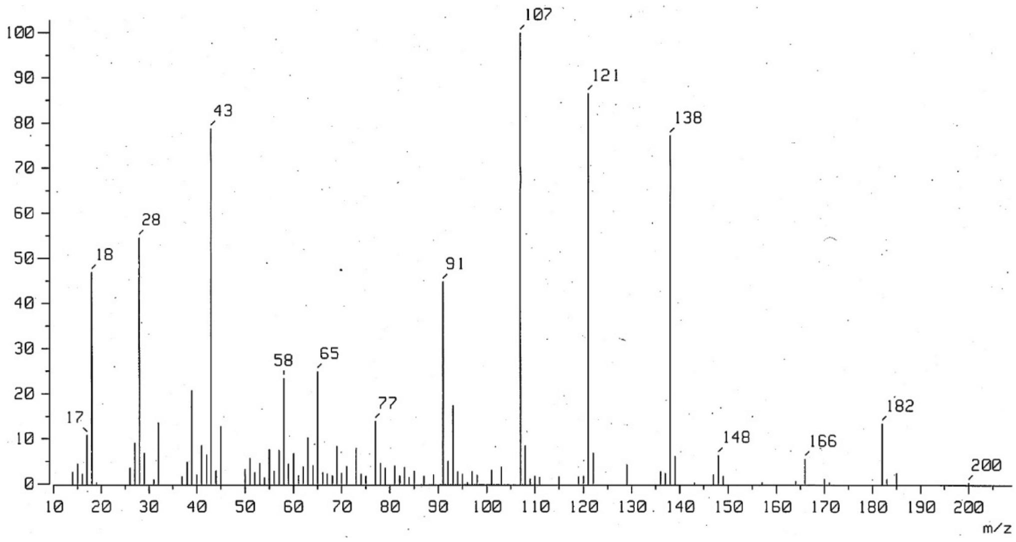

(f)

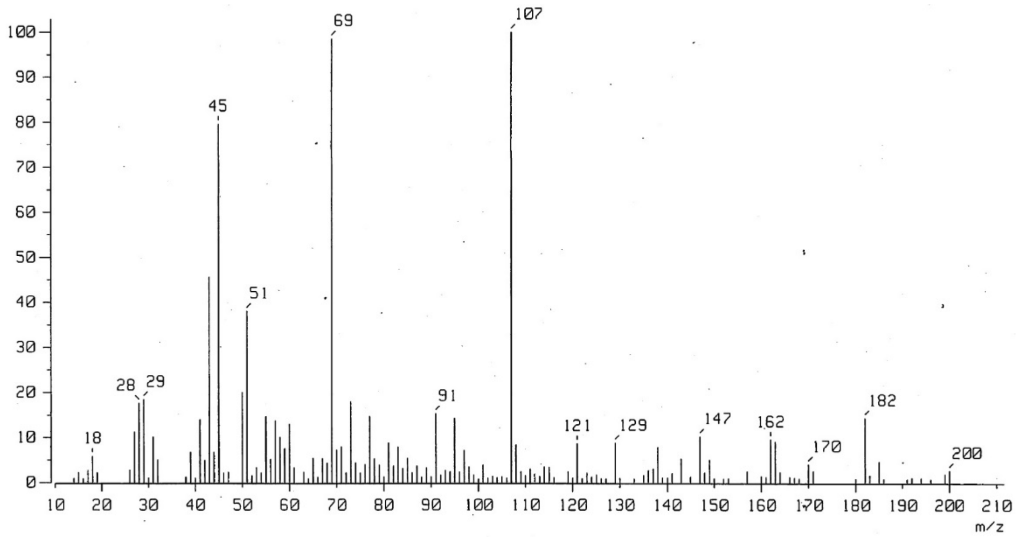

Supplementary Figure S4. FAB-MS (a, b) and EI-MS (c-j) spectra of standard chemicals (a, c, e, g, i) and fractions of HPLC peaks (b, d, f, h, j) described in Figure 5. Standards: (a) DL-Lactic acid (m/z = 91.03, $[M + H]^+$); (c) acetic acid (m/z = 60, $[M]^+$); (e) 4-Hydroxyphenyllactic acid (m/z = 182, $[M]^+$); (g) 4-Hydroxybenzoic acid (m/z = 138, $[M]^+$); (i) 3-Phenyllactic acid (m/z = 166, $[M]^+$). Samples: (b) peak A (m/z = 91.04, $[M + H]^+$); (d) peak B (m/z = 60, $[M]^+$); (f) peak C (m/z = 182, $[M]^+$); (h) peak D (m/z = 138, $[M]^+$); and (j) peak E (m/z = 166, $[M]^+$).

(g)

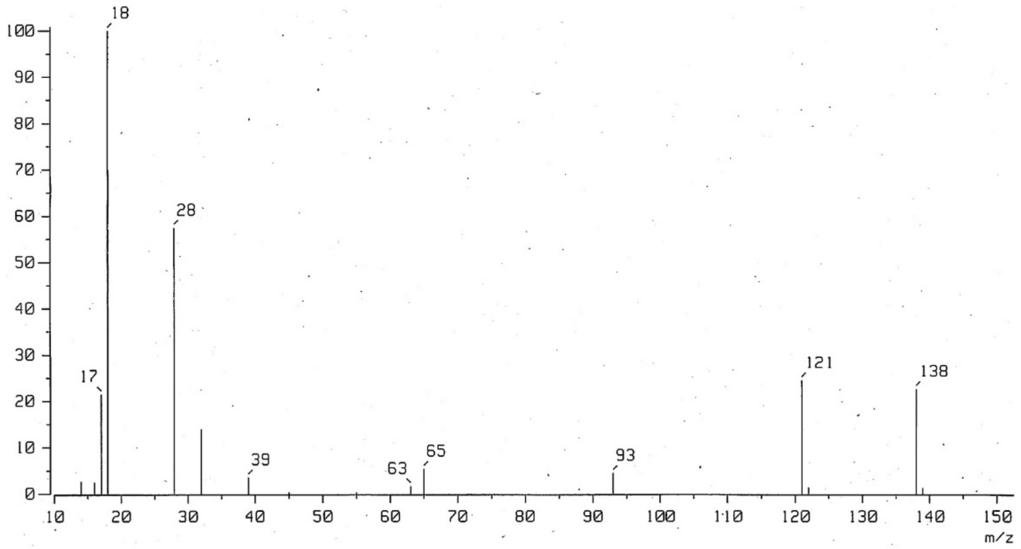

(h)

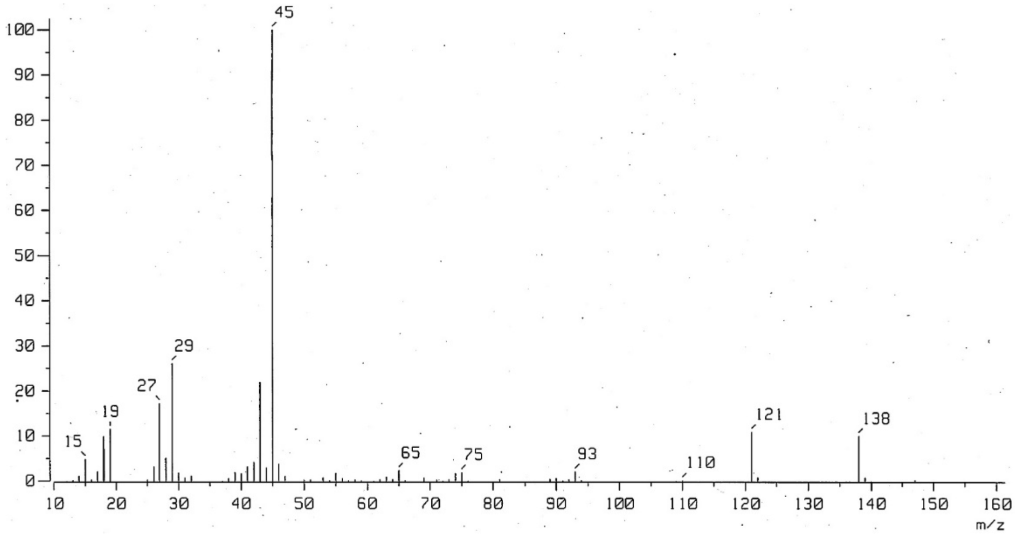

Supplementary Figure S4. FAB-MS (a, b) and EI-MS (c-j) spectra of standard chemicals (a, c, e, g, i) and fractions of HPLC peaks (b, d, f, h, j) described in Figure 5. Standards: (a) DL-Lactic acid (m/z = 91.03, $[M + H]^+$); (c) acetic acid (m/z = 60, $[M]^+$); (e) 4-Hydroxyphenyllactic acid (m/z = 182, $[M]^+$); (g) 4-Hydroxybenzoic acid (m/z = 138, $[M]^+$); (i) 3-Phenyllactic acid (m/z = 166, $[M]^+$). Samples: (b) peak A (m/z = 91.04, $[M + H]^+$); (d) peak B (m/z = 60, $[M]^+$); (f) peak C (m/z = 182, $[M]^+$); (h) peak D (m/z = 138, $[M]^+$); and (j) peak E (m/z = 166, $[M]^+$).

(i)

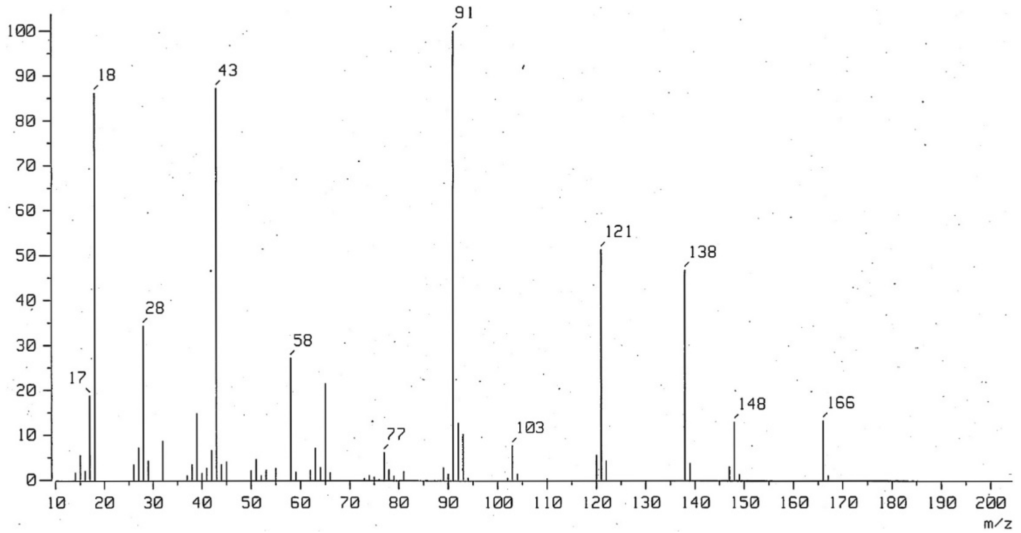

(j)

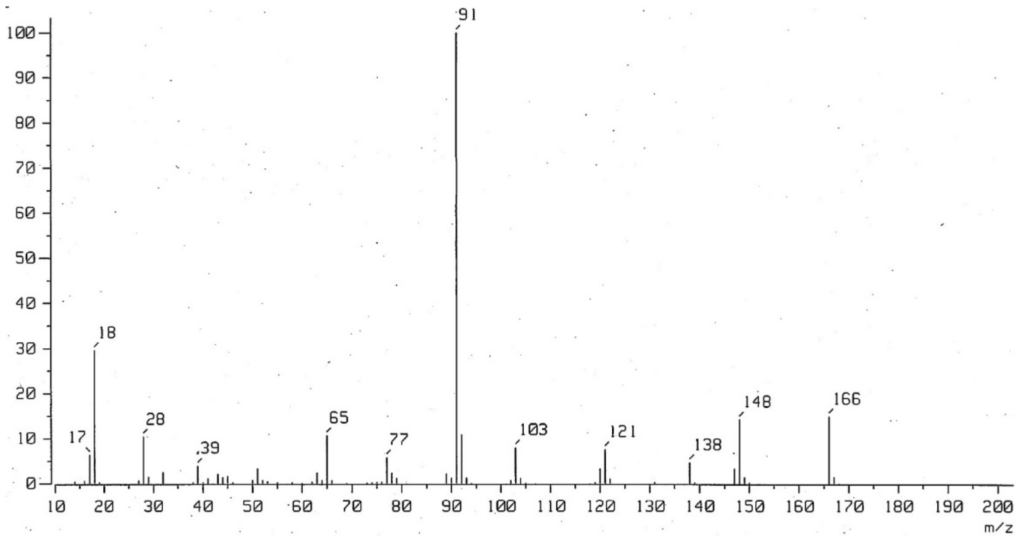

Supplementary Figure S4. FAB-MS (a, b) and EI-MS (c-j) spectra of standard chemicals (a, c, e, g, i) and fractions of HPLC peaks (b, d, f, h, j) described in Figure 5. Standards: (a) DL-Lactic acid (m/z = 91.03, $[M + H]^+$); (c) acetic acid (m/z = 60, $[M]^+$); (e) 4-Hydroxyphenyllactic acid (m/z = 182, $[M]^+$); (g) 4-Hydroxybenzoic acid (m/z = 138, $[M]^+$); (i) 3-Phenyllactic acid (m/z = 166, $[M]^+$). Samples: (b) peak A (m/z = 91.04, $[M + H]^+$); (d) peak B (m/z = 60, $[M]^+$); (f) peak C (m/z = 182, $[M]^+$); (h) peak D (m/z = 138, $[M]^+$); and (j) peak E (m/z = 166, $[M]^+$).

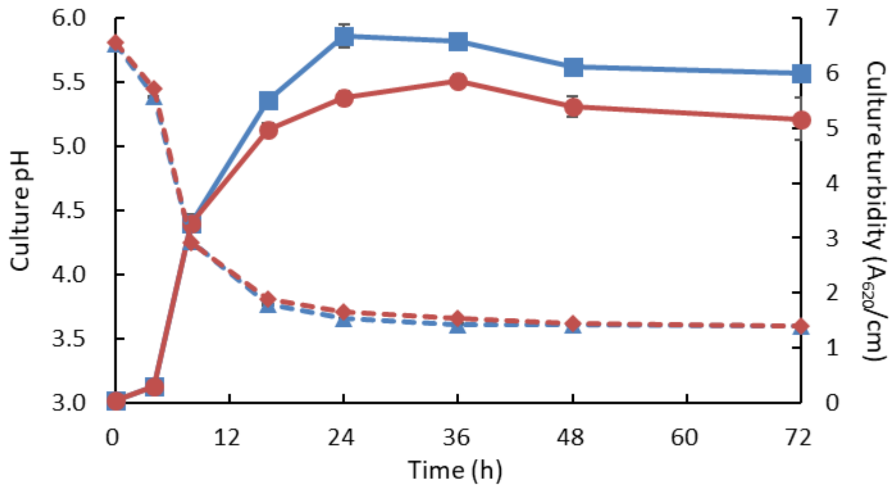

Supplementary Figure S5. Changes in culture pH (broken lines) and turbidity (solid lines) of *Lactiplantibacillus plantarum* 3121M0s (light blue) and JCM 1149^{T} (red) cultivated in MRS broth at 30°C for 72 h.
